# Effects of Substituents on 9‑Fluorenone: Spectroscopic, Hirshfeld, Optical, NLO, IFCT, and CTM Properties

**DOI:** 10.1021/acsomega.5c13532

**Published:** 2026-03-12

**Authors:** Feride Akman

**Affiliations:** † Vocational School of Food, Agriculture and Livestock, Bingol University, Bingol 12000, Turkey; ‡ Chemistry Programme, Institute of Sciences, Bingol University, Bingol 12000, Turkey

## Abstract

The effect of substituent groups on 9-fluorenone derivatives was investigated using density functional theory (DFT) and the Multi-Objective Wave Function Analyzer for Chemists. 9-Fluorenone derivatives, substituted at the 2-position, were studied in terms of their structural, electronic, and optical properties. The effects of electron-donating and electron-withdrawing groups were investigated through FTIR and ^1^H NMR spectra, and the absorption and emission spectra were determined to identify electronic transitions and optical properties. For the electronic properties of the studied molecules, HOMO–LUMO molecular orbital analyses were performed, and average local ionization energy (ALIE) and electrostatic potential (ESP) surface analyses were conducted to determine the reactive regions of the molecules. Additionally, electron density-based analyses such as ELF, CTM, LOL, IFCT, and LOLIPOP were also carried out. The effect of substituent groups at the 2-position of 9-fluorenone on the dipole moment, polarizability, and nonlinear optical (NLO) properties was evaluated, and it was found that the 2-nitro-9-fluorenone molecule exhibited higher charge transfer. Harmonic Oscillator Model of Aromaticity (HOMA) index was determined to assess aromaticity. Finally, crystal packing and Hirshfeld surfaces were determined. The results suggest that the studied 9-fluorenone derivatives, with their electronic polarization, emission, absorption and nonlinear optical (NLO) properties, are potential candidates for applications such as organic field-effect transistors (OFET), liquid crystals, optical brighteners, organic photovoltaics (OPV), organic light-emitting diodes (OLED) and similar applications, and that other derivatives may also be developed.

## Graphical abstract

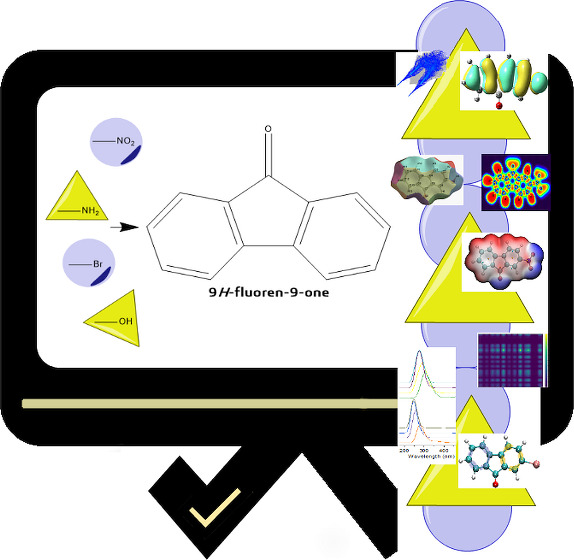

## 1 Introduction

9-Fluorenone or 9H-Fluoren-9-one (dibenzopentacyclic ketone) is a common byproduct of coal tar deep processing and is used as an important raw material in fine chemical synthesis, and it is mostly used in the preparation of dyes, resin modification, and material additives.^1,2^ Owing to its rigid and planar fused aromatic ring system containing a carbonyl group, the fluorenone framework exhibits remarkable photophysical and optoelectronic properties, making it a valuable structural motif in the design of advanced functional materials.^3,4^ Due to their extended π-conjugation, the chemical and thermal stability and resistance to air, and electron-accepting characteristics, fluorenone derivatives are crucial in materials science, especially in creating organic semiconductors and optoelectronic devices like organic field-effect transistors (OFET), liquid crystals, organic light-emitting diodes (OLED), organic photovoltaics (OPV), and optical brighteners.^3–10^ Recent studies have demonstrated the versatility of the fluorenone ring in material applications and its integration on high-efficiency dye-sensitized solar cells^2,11^ and use as a core structure in organic semiconductors.^9,10,12^ Although reports exist on fluorenone-based organic semiconductors, their applications have been primarily investigated in photovoltaic systems. However, due to its rigid and planar structure, strong π-conjugation, and electron-withdrawing properties, fluorenone can enhance charge transport and intramolecular charge transfer processes. This makes it a promising candidate for high-performance organic field-effect transistor (OFET) devices. Therefore, the development of fluorenone derivatives is of great importance for the design of highly efficient, stable, and reliable organic transistors.^9,10^ Fluorenone is notable for its simple electron-deficient structure, high thermal stability, relatively planar geometry, and efficient electron transfer capabilities. Its low cost and easy commercial availability also make it an attractive starting material. Due to its inherent electron deficiency, it is regarded as a promising candidate for designing donor–acceptor (D–A) materials.^9,10^ Moreover, its electronic structure and charge distribution are strongly affected by substituents and the surrounding solvent environment,^13,14^ which makes the molecule highly responsive to structural modifications. Because, substituent groups attached to the fluorenone ring can play an important role in adjusting their photophysical and electronic properties. These substituent groups can affect the electronic distribution of the core structure, emission and absorption properties, adjust energy levels, and alter charge transfer behavior.^13,15^ The specific behavior of the 2-position derivatives encouraged us to explore how electron-withdrawing and electron-donating groups at this position influence fluorenone.^13,15^ The insights gained from this study are expected to provide a scientific basis for the design of new derivatives. For this purpose, we selected classical groups with well-known electron effects and Hammett σ_p_ values,^16^ such as −OH, −NH_2_, −NO_2_, and −Br. These derivatives significantly influence intramolecular charge transfer (ICT) and nonlinear optical (NLO) properties, making them promising candidates for advanced photonic, optoelectronic, and sensing technologies.^17^ Their effects on the 2-position of the fluorenone ring were also investigated. To better understand these effects at the molecular level, computational methods provide a reliable and detailed approach. Therefore, the computational approaches like density functional theory (DFT) and time-dependent DFT (TD-DFT) were used for the structure–property relationships of the studied molecules.^18,19^ That is, we have theoretically investigated four 2-substituted fluorenone derivatives, such as 2-bromo-9-fluorenone, 2-nitro-9-fluorenone, 2-amino-9-fluorenone, and 2-hydroxy-9-fluorenone to explore the effects of different substituents on their electronic, optical, and NLO properties. The findings are expected to contribute to a deeper understanding of the photophysical and photochemical behavior of fluorenone-containing molecular systems and support the design of high-performance materials for optoelectronic and photonic applications. A comprehensive theoretical study was carried out using DFT, which is widely used and reliable method for predicting properties that closely with experimental results,^20,21^ to investigate the effect of substituents on molecular structure and the properties of 2- position of 9-fluorenone. First, after the molecular structure was optimized, theoretical spectroscopic analyses were performed as follows: FTIR spectra were calculated to characterize vibrational modes; chemical shifts of hydrogen atoms were obtained using the GIAO method, and UV–vis absorption spectra were calculated using TD-DFT. Additionally, fluorescence spectra were predicted to evaluate electronic transitions and photophysical behavior. In addition to molecular structure analyses, ESP and ALIE surfaces, HOMO–LUMO energies, and other global chemical reactivity descriptors were determined to understand the electronic and reactive region properties of these molecules. Furthermore, NLO, IFCT, CTM, and LOLIPOP analyses were performed to investigate electron densities, while intermolecular interactions were studied using crystal packing and Hirshfeld surface analysis. As a result, this study provides a comprehensive theoretical understanding of how different substituent groups affect the spectroscopic, photophysical, electronic, and crystal surface properties of fluorenone derivatives. Furthermore, this information is expected to provide useful information for experiments and potential applications in optoelectronic or photonic materials.

## 2 Computational Details

The crystallographic structures of 2-nitro-9-fluorenone (2-NO_2_-9-Fl), 2-amino-9-fluorenone (2-NH_2_-9-Fl), 2-bromo-9-fluorenone (2-Br-9-Fl) and 2-hydroxy-9-fluorenone (2-OH-9-Fl) were obtained from CCDC under reference numbers 715819, 801750, 1986219 and 2033316, respectively.^22^ The crystal packing and ORTEP-3 diagrams of 2-NO_2_-9-Fl, 2-NH_2_-9-Fl, 2-Br-9-Fl and 2-OH-9-Fl were determined using the free Mercury^23^ and ORTEP-3 ^24^ programs, respectively. All quantum chemical calculations were performed using Gaussian 16 and Gaussian 09W.^25^ Geometry optimizations and frequency calculations were carried out using Gaussian 09W at the B3LYP/6–311++G­(d,p) level of theory, starting from the X-ray crystallographic geometries. The B3LYP functional consists of the Becke^26^ three-parameter hybrid exchange functional combined with the Lee–Yang–Parr correlation functional.^27^ The obtained results were visualized using GaussView 5.0.^28^ The chemical shifts of the protons were determined using G16 within the GIAO approach.^29^ The TD-DFT method was used for the UV–vis and fluorescence properties. Multiwfn 3.8 ^30^ and VMD 1.9.3 ^31^ programs were used to calculate electron density-based analyses such as NLO, ELF, CTM, IFCT, LOLIPOP, and LOL. Crystal Explorer 17.5 software^32^ was used to determine the intermolecular interactions of 2-NO_2_–9-Fl, 2-NH_2_–9-Fl, 2-Br-9-Fl and 2-OH-9-Fl and for Hirshfeld surface analysis.

## 3 Results and Discussion

### 3.1 Optimized Molecular Geometry

The optimized molecular structures of 2-nitro-, 2-amino-, 2-bromo-, and 2-hydroxy- 9-fluorenone derivatives are shown in Figure 1 along with their ORTEP-3 (left) diagrams. Additionally, the atomic numbers and relevant geometric parameters are listed in Table 1. Theoretical calculations of bond lengths were performed under gas phase, and the bond length within the molecule is influenced with orbital hybridization, bond order, and resonance or delocalization of π-electrons.^33,34^

**Figure 1:**
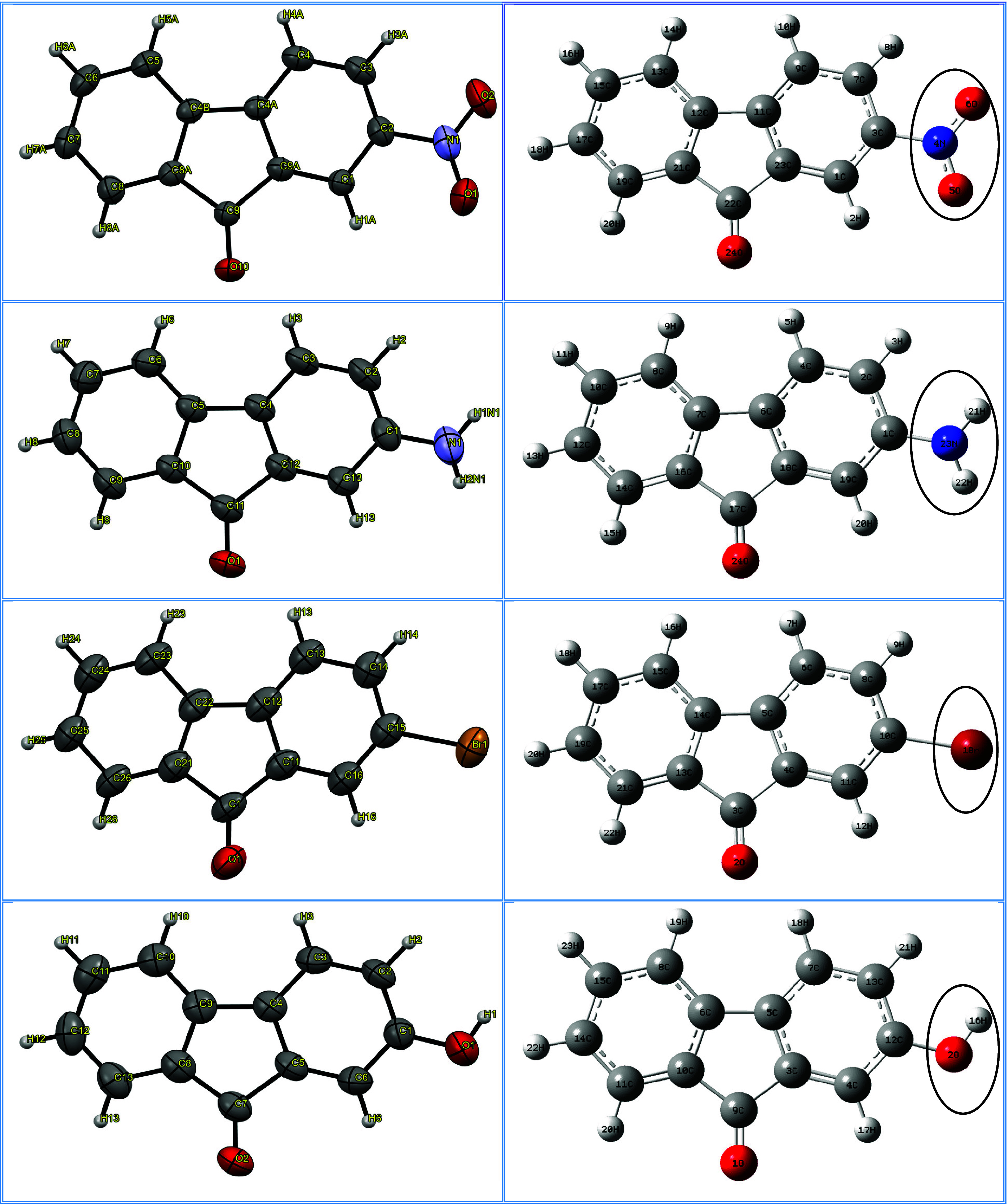
ORTEP-3 (left) and optimized molecular structures (right) of 2-NO_2_-9-Fl, 2-NH_2_-9-Fl, 2-Br-9-Fl and 2-OH-9-Fl.

**Table 1:** Selected Experimental (from Crystal Structures) and Theoretical* (from Optimized Molecular Structures) Bond Parameters of the Studied Fluorenone Derivatives^a^

bond	2-NO_2_-9-Fl	2-NO_2_-9-Fl*	bond	2-NH_2_-9-Fl	2-NH_2_-9-Fl*	bond	2-Br-9-Fl	2-Br-9-Fl*	bond	2-OH-9-Fl	2-OH-9-Fl*
C1–C2 (C1–C3)	1.398(3)	1.397	C1–C13 (C1–C19)	1.401(2)	1.4088	C15–C16 (C10–C11)	1.38(2)	1.3969	C6–C1 (C4–C12)	1.398(3)	1.4018
C2–C3 (C3–C7)	1.383(3)	1.3935	C1–C2 (C1–C2)	1.393(2)	1.4056	C14–C15 (C8–C10)	1.40(2)	1.3939	C1–C2 (C12–C13)	1.395(3)	1.3977
C3–C4 (C7–C9)	1.391(3)	1.3961	C2–C3(C2–C4)	1.382(2)	1.3969	C13–C14 (C6–C8)	1.40(2)	1.4002	C2–C3 (C7–C13)	1.392(3)	1.4002
C1–C9A (C1–C23)	1.372(3)	1.3811	C12–C13 (C18–C19)	1.376(2)	1.3803	C11–C16 (C4–C11)	1.39(2)	1.3843	C5–C6 (C3–C4)	1.376(3)	1.3808
C4–C4A (C9–C11)	1.388(3)	1.3905	C3–C4 (C4–C6)	1.383(2)	1.3885	C12–C13 (C5–C6)	1.39(2)	1.3882	C3–C4 (C5–C7)	1.383(3)	1.3868
C9–O10 (C22–O24)	1.211(2)	1.21	C11–O1 (C17–O24)	1.220(1)	1.2129	C1–O1 (C3–O2)	1.22(2)	1.2112	C7–O2 (C9–O1)	1.222(3)	1.2119
C4A–C4B(C11–C12)	1.481(2)	1.48	C4–C5 (C6–C7)	1.475(2)	1.4796	C12–C22 (C5–C14)	1.48(2)	1.4821	C4–C9 (C5–C6)	1.479(3)	1.481
C2–N1 (C3–N4)	1.471(3)	1.4776	C1–N1 (C1–N23)	1.381(2)	1.3927	C15–Br1 (C10–Br1)	1.90(1)	1.916	C1–O1 (C12–O2)	1.352(3)	1.3667
C1–C2–C3 (C1–C3–C7)	123.0(2)	122.4525	C2–C1–C13 (C2–C1–C19)	118.5(1)	118.8807	C14–C15–C16 (C8–C10–C11)	122(1)	121.5939	C2–C1–C6 (C4–C12–C13)	120.4(2)	120.4331
C8–C9–C9A (C21–C22–C23)	105.1(1)	104.7857	C10–C11–C12 (C16–C17–C18)	105.6(1)	104.9414	C11–C1–C21 (C4–C3–C13)	105(1)	104.8332	C5–C7–C8 (C3–C9–C10)	105.7(2)	104.9257

a Atomic numbering as in Figure 1; parentheses indicate optimized, outside indicate crystal structure. Bond parameters of equivalent bonds in each molecule are given in the same row.

The geometries of four fluorenone derivatives (2-NO_2_-9-Fl, 2-NH_2_-9-Fl, 2-Br-9-Fl and 2-OH-9-Fl) were optimized using the B3LYP/6–311++G­(d,p) method, and the initial experimental parameters from their corresponding crystal structures, used for comparison, are summarized in Table 1. In Table 1, the atomic numbering follows Figure 1, and equivalent bond lengths and angles in each molecule are listed in the same row for direct comparison. To facilitate the comparison of the experimental and theoretical bond lengths given in Table 1. For comparison, the data are presented as a column graph in Figure 2. The carbonyl bonds (C=O) in 2-NO_2_-9-Fl (C9–O10 (C22–O24), 1.211 Å in crystal structure, 1.21 Å in optimized structure), 2-NH_2_-9-Fl (C11–O1 (C17–O24), 1.220 Å in crystal structure, 1.2129 Å in optimized structure), 2-Br-9-Fl (C1–O1 (C3–O2), 1.22 Å in crystal structure, 1.2112 Å in optimized structure), and 2-OH-9-Fl (C7–O2 (C9–O1), 1.222 Å in crystal structure, 1.2119 Å in optimized structure) show minimal deviations upon optimization upon optimization, confirming their strong double-bond character, although slight variations are observed depending on the nature of the substituent. Slight variations in bond lengths (1.37–1.41 Å) are observed in the aromatic ring depending on the nature of the substituent. The electron-withdrawing −NO_2_ group, attached to the carbon atom bonded to the substituent, slightly lengthens the C–N bond to 1.471 (1.4776) Å, whereas the electron-donating −NH_2_ group shortens this bond to 1.381 (1.3927) Å due to enhanced conjugation with the aromatic ring. This trend also parallels the Hammett σ_p_ parameters of the substituents.^16^ Hammett values are considered negative for electron-donating groups (EDG), positive for electron-withdrawing groups (EWG), and zero for hydrogen atoms.^16^ It can be said that the interaction of the −NH_2_ group with a negative σ value strengthens it, while the interaction of the −NO_2_ group with a positive σ value weakens it. Therefore, the change in the C–N bond length appears to be related to the electronic effect of the substituents.^16,35^ This effect is also observed in the bonds C4A-C4B (C11–C12) with 1.481 and 1.48 Å, C4–C5 (C6–C7) with 1.475(2) and 1.4796 Å, C12–C22 (C5–C14) with 1.48 and 1.4821 Å, and C4–C9 (C5–C6) with 1.479 and 1.481 Å, showing similar substituent-dependent variations in the five-membered ring. The results indicate that the optimized structures with DFT are in close agreement with the experimental crystal geometries and that the effects of the substituents are clearly observable. According to the literature review, the carbonyl C=O bond length in unsubstituted 9-fluorenone has been experimentally determined to be 1.220 Å by X-ray crystallography.^36^ In DFT study on fluorenone, Peng Song et al.,^4^ reported that the C=O bond in the ground state of fluorenone is 1.212 Å and elongates to 1.250 Å upon electronic excitation. Similarly, Kawabata et al.,^37^ calculated the C=O bond length of free fluorenone to be 1.2127 Å at the B3LYP/6–311+G­(d) level and observed it to elongate to 1.2828 Å upon interaction with a sodium atom. The results from these experimental and computational studies are compatible with both the crystal structure and theoretical calculations. Moreover, the RMSD values between the selected experimental (from crystal structures) and theoretical (from optimized molecular structures) bond lengths of the fluorenone derivatives examined in Table 1 were calculated and found to be 0.0058 Å for 2-NO_2_-9-Fl, 0.0093 Å for 2-NH_2_-9-Fl, 0.0093 Å for 2-Br-9-Fl, and 0.0075 Å for 2-OH-9-Fl, respectively. In general, hydrogen bonds, π–π interactions and crystal packing forces can slightly modify experimental bond lengths by compressing or stretching bonds compared to isolated gas-phase geometries.^38,39^ Despite these effects, optimized gas-phase geometries are generally reported to be in good agreement with crystallographic data, with bond length differences of approximately 0.01–0.02 Å and bond angles within approximately 1°.^21,40^ Furthermore, hybrid GGA functionals are known to provide low mean errors in bond length calculations and reliable agreement with experimental structures.^21^ Therefore, although crystallographic effects may cause small deviations, computationally optimized structures are considered to be a reliable representation of the molecule’s intrinsic geometry.^38,39^ Besides, the RMSD values between optimized and experimental bond lengths (the bond lengths selected in Table 1 were considered) for different substituents were calculated. Based on these results, we can say that DFT calculations have been successful in determining our molecular geometry.

**Figure 2:**
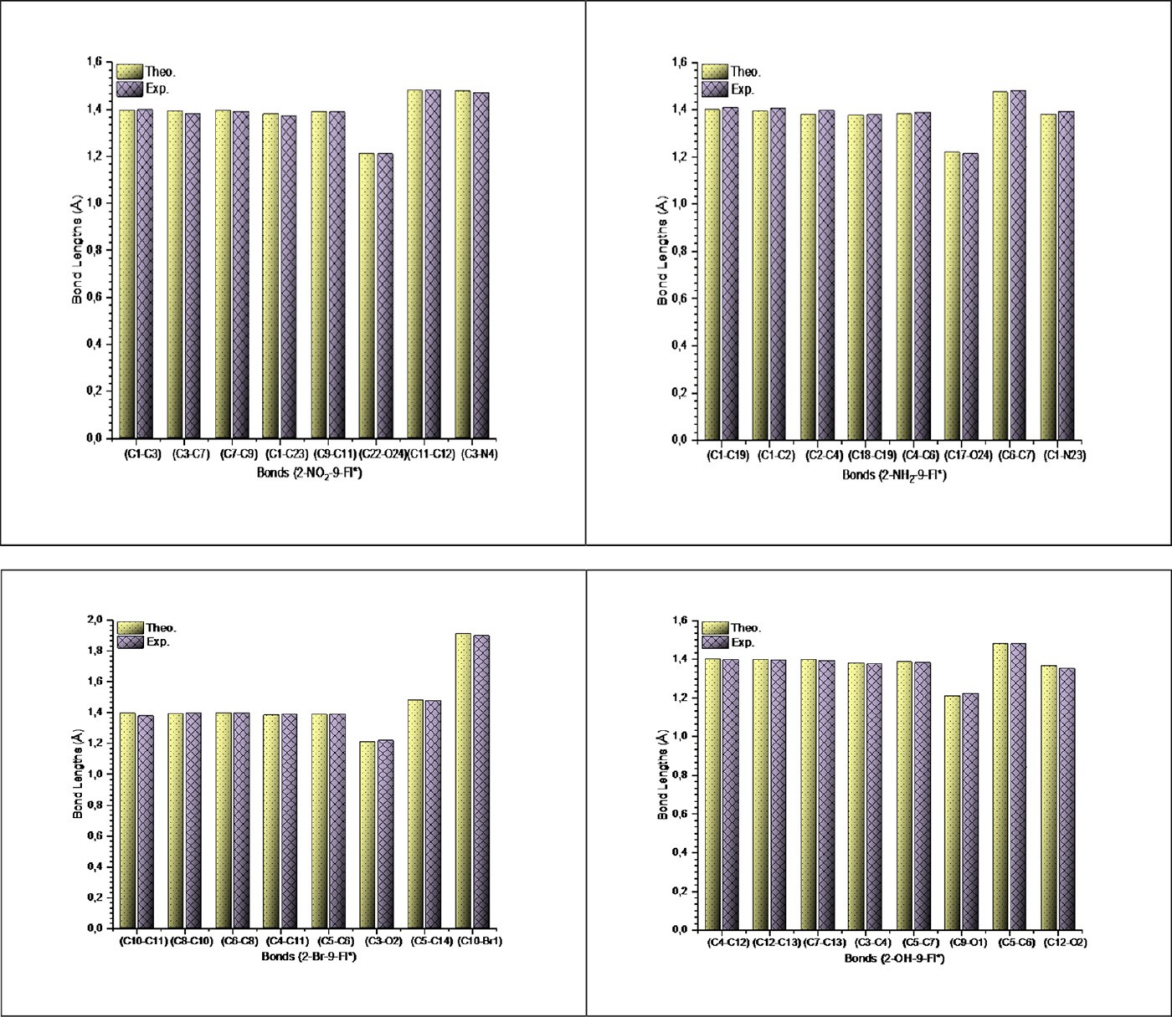
A comparison chart of experimental and theoretical bond lengths (Å) for 2-NO_2_-9-Fl, 2-NH_2_-9-Fl, 2-Br-9-Fl and 2-OH-9-Fl.

### 3.2 Vibrational Analysis

To investigate the effect of substituents on FTIR spectra, the theoretical FTIR spectra of 2-NO_2_-9-Fl, 2-NH_2_-9-Fl, 2-Br-9-Fl and 2-OH-9-Fl were calculated and compared with each other and are shown in Figure 3. According to the obtained results, all compounds exhibited characteristic absorption bands corresponding to the fundamental functional groups of the fluorenone backbone, namely C=O, aromatic C=C, and aromatic C–H stretching vibrations. In addition, characteristic bands specific to the substituent groups were also observed. A scaling factor of 0.9668 cm^–1^ (https://www.cccbdb.nist.gov/vsfx.asp) was applied in accordance with the basis set used in the calculations. In 2-NO_2_-9-Fl, the aromatic C–H stretching vibrations were observed in the 3114–3064 cm^–1^ region. Aromatic C=C stretching vibrations appeared in the 1595–1292 cm^–1^ region, while a strong C=O stretching band was observed around 1729 cm^–1^. The asymmetric N=O stretch appears at 1529 cm^–1^, and the symmetric N=O stretch, coupled with aromatic C–N stretching, appears at 1318 cm^–1^. In 2-NH_2_-9-Fl, the aromatic C–H stretching vibrations were also found in the 3086–3053 cm^–1^ region. Aromatic C=C stretching bands were observed in the 1610–1279 cm^–1^ region, and a strong C=O stretching vibration appeared near 1717 cm^–1^. Additionally, the asymmetric and symmetric N–H stretching vibrations were observed at 3554 cm^–1^ and 3457 cm^–1^. In 2-Br-9-Fl, the aromatic C–H stretching vibrations appeared in the 3097–3062 cm^–1^ region, while aromatic C=C stretching vibrations were found in the 1590–1272 cm^–1^ region. The C=O stretching vibration showed a strong band at around 1723 cm^–1^. The C–Br stretching band was observed at around 451 cm^–1^. In 2-OH-9-Fl, the aromatic C–H stretching vibrations were found in the 3091–3048 cm^–1^ region, while aromatic C=C stretching vibrations appeared in the 1601–1289 cm^–1^ region, and the C=O stretching vibration was observed around 1721 cm^–1^. The O–H stretching band appeared at 3705 cm^–1^. The effect of substituents on the C=O stretching vibration was clearly observed. The order of C=O stretching frequencies from highest to lowest is as follows: 2-nitro > 2-bromo > 2-hydroxy > 2-amino-9-fluorenone. This trend is compatible with the electron-withdrawing and electron-donating nature of the substituents. Electron-withdrawing groups (such as −NO_2_ and −Br) increase the bond strength of the carbonyl group, leading to higher C=O stretching frequencies, whereas electron-donating groups (such as −OH and −NH_2_) tend to lower the frequency. In a study examining the IR spectrum of fluorenone under varying electric field (EF) strengths, a distinct peak corresponding to the carbonyl group’s characteristic C=O stretching vibration appears around 1700 cm^–1^ when no electric field is applied (EF = 0).^41^ Similarly, when the FT-IR spectra of the fluorene-fluorenone-fluorene (BFF) trimer model compound were examined together with copolymers of varying ratios, two main vibration bands were observed at 1718 and 1448 cm^–1^, corresponding to the C=O stretching and aromatic C=C stretching of the fluorenone unit. Additionally, a peak at 1606 cm^–1^ was attributed to the stretching mode of the asymmetrically substituted benzene ring within the fluorenone unit. Notably, the relative intensity of the keto vibration band (1718 cm^–1^) increases as the fluorenone content in the copolymers increases.^42^ Moreover, for the compound 9-fluorenone-2-carboxylic acid, the experimental C=O band was observed at 1718 cm^–1^ in the FT-IR analysis. While the scaled theoretical frequency reported in the literature using the B3LYP/6–31G­(d,p) method is 1741 cm^–1^.^43^ These findings are compatible with the FT-IR results calculated in our study.

**Figure 3:**
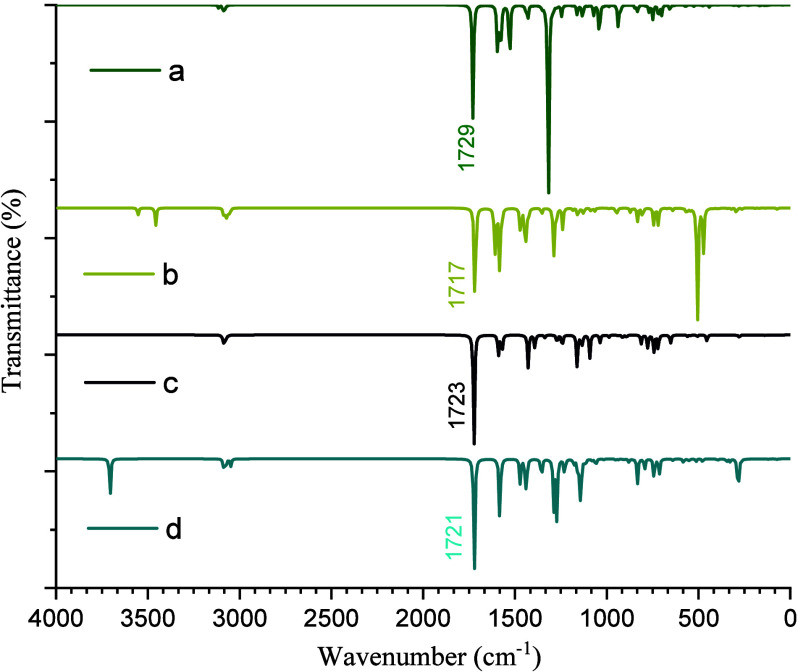
Theoretical FTIR spectra of 2-NO_2_-9-Fl (a), 2-NH_2_-9-Fl (b), 2-Br-9-Fl (c), and 2-OH-9-Fl (d) scaled by 0.9668.

### 3.3 Nuclear Magnetic Resonance Analysis

The ^1^H NMR chemical shifts of fluorenone derivatives such as 2-NO_2_-9-Fl, 2-NH_2_-9-Fl, 2-Br-9-Fl and 2-OH-9-Fl were calculated using the GIAO method at the CAM-B3LYP-D3/6–311++G­(d,p) level in CPCM/DCM in a solvent system and are illustrated in Figure 4. The aromatic protons exhibit distinct chemical shift ranges depending on the nature of the substituent: 7.80–8.90 ppm for the electron-withdrawing −NO_2_ group, 7.10–7.95 ppm for the electron-donating −NH_2_ group, 7.64–8.08 ppm for the moderately withdrawing −Br substituent, and 7.12–8.00 ppm for the −OH group, which is a weakly electron-donating substituent. Notably, the protons (H21 and H22) attached to the amino group in 2-NH_2_-9-Fl resonate at 3.76 and 3.75 ppm, respectively. For 2-OH-9-Fl, the signal at 4.71 ppm is related to a proton of the hydroxyl group attached to the fluorenone ring. The results indicate that the chemical shifts of the aromatic protons in the fluorenone ring depend on the nature of the attached substituents. In particular, the presence of electron-withdrawing groups such as −NO_2_ and −Br groups causes the protons to shift to higher ppm values (downfield), whereas electron-donating groups like −NH_2_ and −OH shift the protons to lower ppm values (upfield).

**Figure 4:**
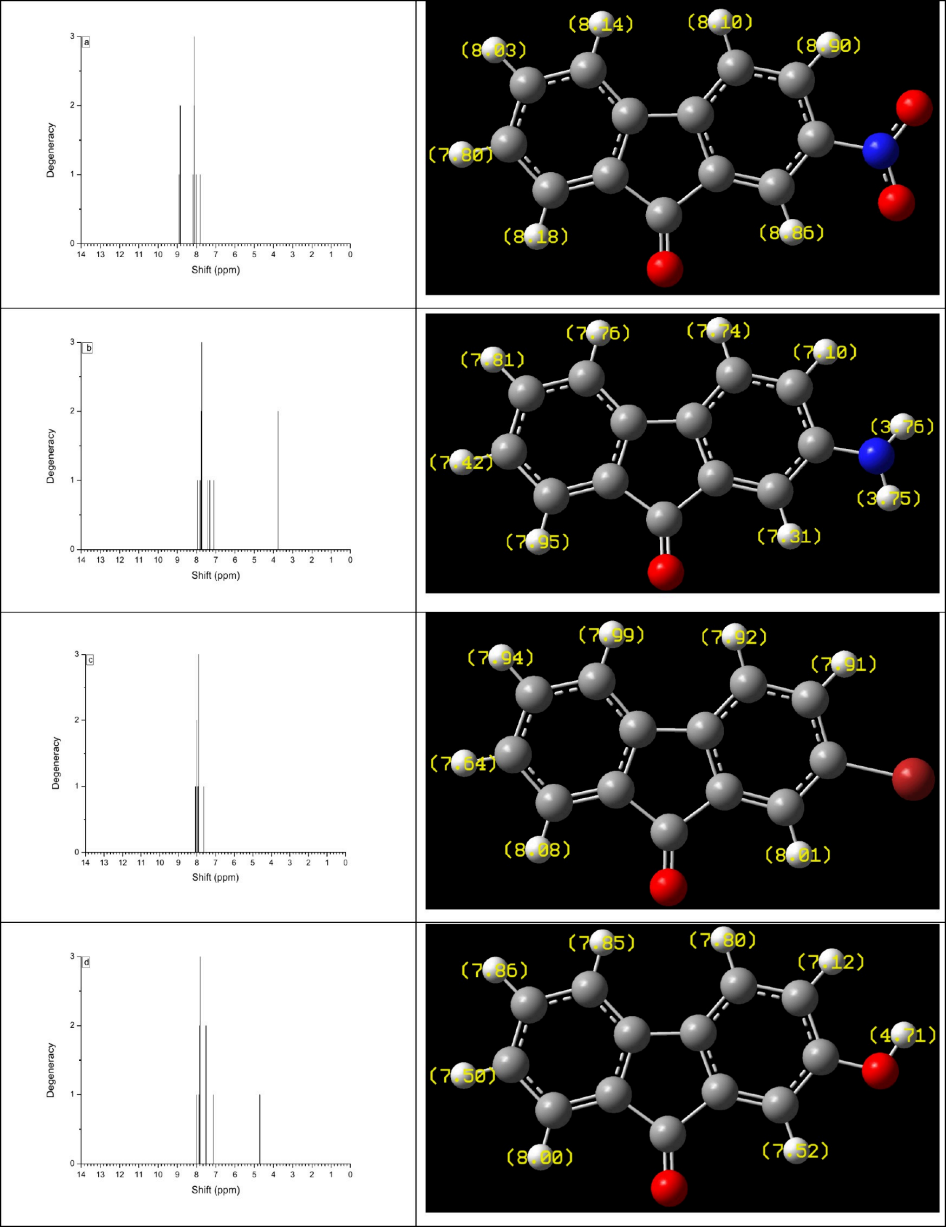
Theoretical ^1^H NMR spectra of 2-NO_2_-9-Fl (a), 2-NH_2_-9-Fl (b), 2-Br-9-Fl (c), and 2-OH-9-Fl (d) in CPCM/DCM.

According to the literature, the ^1^H NMR spectra of copolymers with different ratios of the fluorene-fluorenone-fluorene (BFF) trimer model compound show that the chemical shifts of the protons in the aromatic ring of the fluorenone unit are in the range of 7.6–8.1 ppm.^41^ Moreover, in a study of the 9-fluorenone-2-carboxylic acid compound, with protons H18, H19, and H20 in the aromatic ring on the side to which the carboxylic acid is attached, the theoretical values calculated using the B3LYP/6–31G­(d) method in DMSO solvent were 9.184, 8.916, and 8.823 ppm, respectively, and the experimental values were 7.906, 8.012, and 7.880 ppm. The other aromatic ring protons H21, H22, H23, and H24 show theoretical values of 8.815, 8.738, 8.550, and 8.820 ppm, respectively, and experimental values of 7.670, 7.670, 7.670, and 7.880 ppm, respectively.^42^ The results show that the method we used in our theoretical calculations is compatible with the experimental data in the literature.

### 3.4 HOMO–LUMO and NBO Analysis

Fluorenone derivatives play an important role in materials science due to their extensive π-conjugation and electron-accepting properties.^43^ The electronic performance of such materials is largely determined by their molecular orbitals, specifically the energies of HOMO and LUMO, as well as the energy gap between them. The changes in the HOMO–LUMO energy gap provide a powerful strategy for controlling semiconducting and photophysical properties.^43–45^ Generally, in the design of organic light-emitting materials, solar cell components, and fluorescent probe molecules, adding electron-donating or electron-withdrawing groups to dye molecules is a frequently used strategy to precisely tune the molecular boundary orbital energies and absorption and emission properties.^46^ Moreover, fluorenone derivatives with electron-donating or electron-withdrawing substituents can modulate orbital energies, thereby controlling the band gap, ionization potential, and overall molecular reactivity.^43,45^ Therefore, the HOMO and LUMO orbital energies and related reactivity parameters of fluorenone derivatives were calculated.^47,48^ The results are shown in Figure 5 and are listed in Table 2. According to Table 2, substitution at position 2 of 9-fluorenone induces significant changes in the electronic structure of the molecule. The HOMO–LUMO energy gap decreases in the following order: 2-nitro > 2-bromo > 2-hydroxy > 2-amino-9-fluorenone. When considering Hammett σ_p_ constants, which reflect the electron-donating or electron-withdrawing nature of substituents, the groups can be roughly ranked from strongest donor to strongest acceptor as −NH_2_, −OH, −Br, and −NO_2_.^16,45^ The substituents at positions 2 of 9-fluorenone interact directly with the carbonyl group via resonance, exhibiting an electronic behavior similar to that of a para-substituted benzoic acid system. Therefore, para Hammett sigma constants (σ_para_), which reflect both inductive and resonance effects, have been preferred in the ranking of substituents.^45^ This ranking generally matches the calculated HOMO–LUMO gaps, with stronger electron-donating groups tending to slightly reduce the gap, making the molecule softer and more reactive, and electron-withdrawing groups tend to increase the gap, leading to greater stability. Similar trends have been reported in the literature for other fluorenone derivatives.^45^ In 2-NO_2_-9-Fl, the strong electron-withdrawing −NO_2_ group significantly stabilizes both the HOMO (−7.24 eV) and LUMO (−3.37 eV) orbitals. This orbital lowering can be attributed to the inductive and resonance effects of the nitro group, which withdraws electron density from the conjugated fluorenone molecule. As a result, this compound exhibits the largest HOMO–LUMO gap (3.87 eV), the highest electrophilicity index (7.28 eV), and the greatest molecular hardness, reflecting a high stability and low reactivity. However, 2-NH_2_-9-Fl shows the opposite trend due to the electron-donating resonance effect of the −NH_2_ group. Through p−π conjugation, the amino group donates electron density to the π-system, raising the HOMO energy from −7.24 eV (nitro derivative) to −5.78 eV and slightly increasing the LUMO energy to −2.49 eV. This orbital elevation reduces the HOMO–LUMO gap to 3.29 eV. The increased HOMO energy correlates with a decreased ionization potential and lower electrophilicity, compatible with the expected behavior of electron-donating groups in molecular orbital theory. 2-Br-9-Fl exhibits intermediate behavior. Bromine exerts both an inductive electron-withdrawing effect and a weak resonance electron-donating effect, resulting in a balanced stabilization of HOMO (−6.62 eV) and LUMO (−2.88 eV). The resulting HOMO–LUMO gap (3.74 eV) and electrophilicity index (6.03 eV) suggest moderate reactivity between the nitro and amino derivatives. Similarly, in 2-OH-9-Fl, the −OH group acts as a electron-donor with π-resonance and can form potential intramolecular hydrogen bonds with the carbonyl oxygen.^45^ These interactions lead to intermediate HOMO (−6.22 eV) and LUMO (−2.63 eV) energies. The HOMO–LUMO gap (3.59 eV) and electrophilicity (5.45 eV) indicate that the 2-OH-9-Fl derivative behaves as an electron-donating compound. That is, the observed trends align with the expected behavior of electron-donating and electron-withdrawing groups: electron-donating groups such as −NH_2_ and −OH increase frontier orbital energies, decrease ionization potential (IP) and electron affinity (EA), whereas electron-withdrawing groups like −NO_2_ and −Br stabilize both orbitals, increasing molecular hardness (η) and electrophilicity (ω). In the literature, the HOMO–LUMO energy levels obtained from electrochemical and optical data were determined as follows for the band gap values of the monosubstituted 9-fluorenone derivatives: 2.86 eV for the −OH derivative, 2.99 eV for the −Br derivative, 2.25 eV for the −NH_2_ derivative, and 3.22 eV for the −NO_2_ derivative. These data show that electron-donating groups (−OH and −NH_2_) increase the HOMO energies and decrease the band gap, while electron-withdrawing group (−NO_2_) decrease the HOMO energies and increase the band gap. On the other hand, bromine substitution provides a moderate band gap value reflecting the combined effects of induction and resonance. This is compatible with the results. In a study on fluorenone, the H–L energy levels obtained from electrochemical and optical data were determined as follows for the band gap values of monosubstituted 9-fluorenone derivatives: 3.22 eV for the −NO_2_ derivative, 2.25 eV for the −NH_2_ derivative, 2.99 eV for the −Br derivative, and 2.86 eV for the −OH derivative. These data show that electron-donating groups (−NH_2_ and −OH) increase the HOMO energies and decrease the band gap, while electron-withdrawing groups (−NO_2_) decrease the HOMO energies and increase the band gap. Furthermore, in a study conducted using the B3LYP/6–31G (d,p) method on 9-fluorenone-2-carboxylic acid, the energy band gap is 4.0492 eV.^42^ According to these results, the calculated parameter values are close to the parameters we calculated in our study. This change of orbital energies arises from the interactions between the substituent and the conjugated π-framework, as also reported in previous theoretical studies on substituted naphthalene derivatives.^46^ Therefore, it is crucial to control the electronic and reactive properties of 9-fluorenone derivatives by adjusting their frontier molecular orbitals.

**Figure 5:**
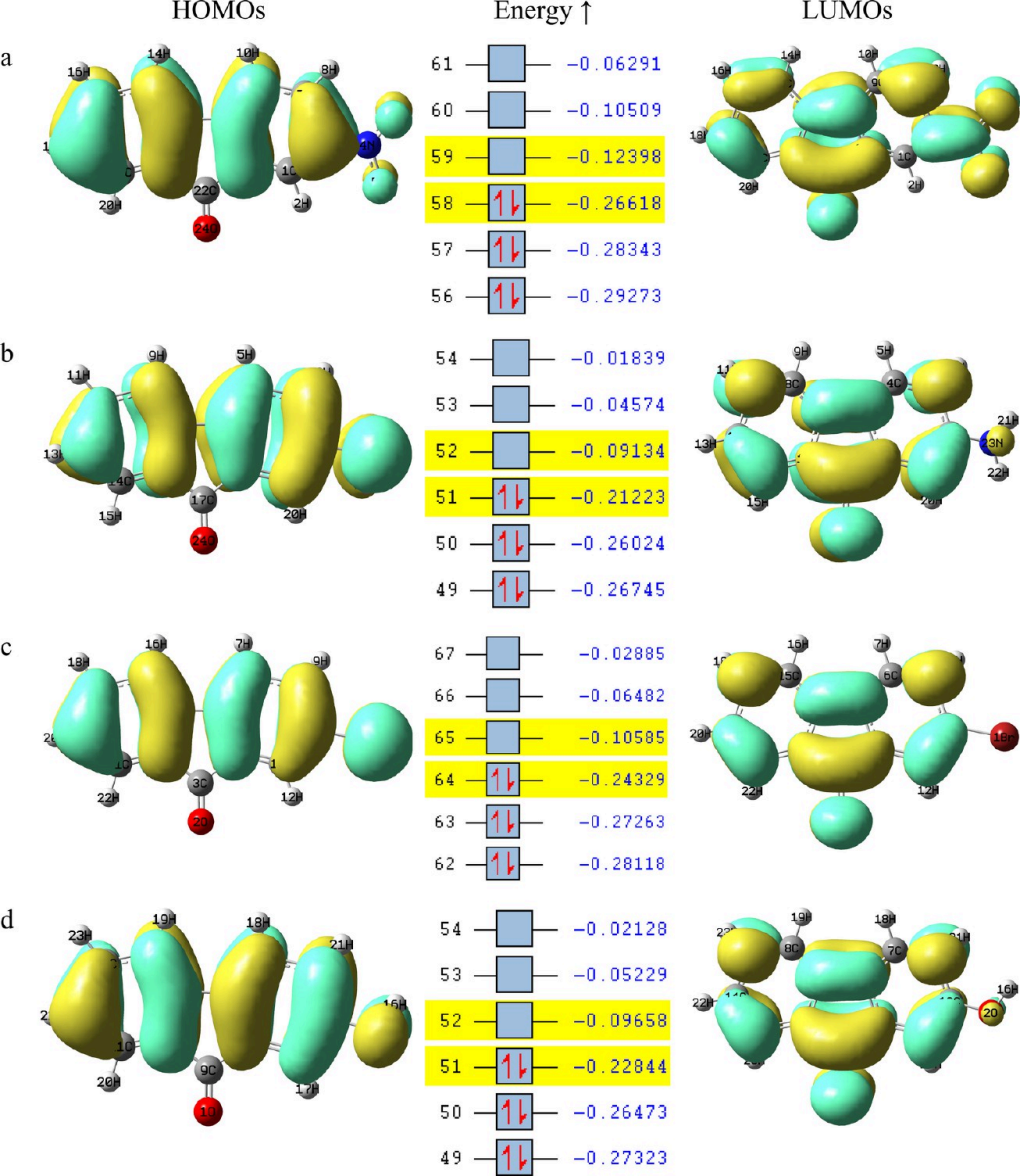
HOMO–LUMO plots of 2-NO_2_-9-Fl (a), 2-NH_2_-9-Fl (b), 2-Br-9-Fl (c), and 2-OH-9-Fl (d) in the ground state.

**Table 2:** HOMO and LUMO Orbital Energies and Related Reactivity Parameters of Fluorenone Derivatives

parameter (eV)	2-NO_2_-9-Fl	2-NH_2_-9-Fl	2-Br-9-Fl	2-OH-9-Fl
*E*_HOMO_	–7.2431	–5.7751	–6.6203	–6.2162
*E*_LUMO_	–3.3737	–2.4855	–2.8803	–2.6281
Δ*E*	3.8695	3.2896	3.7399	3.5881
IP	7.2431	5.7751	6.6203	6.2162
EA	3.3737	2.4855	2.8803	2.6281
χ	5.3084	4.1303	4.7503	4.4221
μ	–5.3084	–4.1303	–4.7503	–4.4221
η	1.9347	1.6448	1.8700	1.7940
ζ	0.5169	0.6080	0.5348	0.5574
ω	7.2824	5.1858	6.0336	5.4500
Δ*N*_max_	2.7437	2.5111	2.5403	2.4649
σ_0_	0.2584	0.3040	0.2674	0.2787
*N*	0.1373	0.1928	0.1657	0.1835

Natural Bond Orbital (NBO) analysis was performed to examine the electron density and bond character in the studied molecules, and calculated using B3LYP/6–311G (d,p). The *E*(^2^) stabilization energies of the selected transitions are listed in Table 3. According to Table 3, the highest energy transitions are as follows: the π (C3–C7) → π*­(N4–O5) for 2-NO2–9-Fl with a stabilization energy of 26.78 kcal/mol, the n (N23) → π*­(C1–C2) for 2-NH2–9-Fl with a stabilization energy of 28.3 kcal/mol, the π (C5–C6) → π*­(C8–C10) for 2-Br-9-Fl with a stabilization energy of 23.28 kcal/mol, and the *n* (O2) → π*­(C12–C13) for 2-OH-9-Fl with a stabilization energy of 28.84 kcal/mol. As shown in Table 1, C–N bond lengths differ in the 2-NO_2_-9-Fl and 2-NH_2_-9-Fl molecules. This trend has previously been noted to be consistent with the Hammett σ_p_ values. NBO analysis also supports this. The calculated *n* → π* stabilization energy of 28.3 kcal/mol in 2-NH_2_-9-Fl indicates delocalization of the nitrogen atom’s lone pair into the π* orbital. Therefore, it can be said that the electron-donating nature of the −NH_2_ group strengthens the *n* → π* interaction and contributes to the shortening of the C–N bond.^35^ Moreover, the lone pair electron density in electronegative atoms weakens the C=O bond because it delocalizes to the π* orbital of the carbonyl group (C=O) (n→π*). The increase in bond length causes a decrease (red shift) in the C=O stretching frequency in the IR spectrum (2-NH_2_–9-Fl and 2-OH-9-Fl). However, despite its strong electron-donating or electron-withdrawing character, the carbonyl bond is inherently strong and geometrically rigid, so substituent effects cause only modest changes in the C=O bond length.^35,49^

**Table 3:** Second Order Perturbation Theory Analysis of the Fock Matrix in the NBO Basis of Fluorenone Derivatives

	donor NBO(i)	type	acceptor NBO(*j*)	type	*E*(^2^) kcal/mol	*E*(*j*)–*E*(*i*) a.u.	*F*(*i*,*j*) a.u.
2-NO_2_-9-Fl	C1–C23	π	C9–C11	π*	22.33	0.29	0.072
	C3–C7	π	C1–C23	π*	20.81	0.31	0.072
	C3–C7	π	N4–O5	π*	26.78	0.15	0.061
	C9–C11	π	C3–C7	π*	24.89	0.28	0.074
	C12–C13	π	C15–C17	π*	20.54	0.28	0.069
	C15–C17	π	C19–C21	π*	20.44	0.29	0.069
	C19–C21	π	C12–C13	π*	21.48	0.29	0.071
	O5	LP(2)	C3–N4	σ*	13.53	0.56	0.078
	O5	LP(2)	N4–O6	σ*	18.71	0.72	0.105
	O6	LP(2)	C3–N4	σ*	13.46	0.56	0.078
	O6	LP(2)	N4–O5	σ*	18.59	0.73	0.105
	O24	LP(2)	C21–C22	σ*	20.72	0.69	0.108
	O24	LP(2)	C22–C23	σ*	21.43	0.68	0.109
2-NH_2_-9-Fl	C1–C2	π	C4–C6	π*	21.02	0.3	0.071
	C4–C6	π	C18–C19	π*	19.34	0.29	0.068
	C7–C8	π	C10–C12	π*	21.21	0.28	0.070
	C10–C12	π	C14–C16	π*	20.03	0.30	0.069
	C14–C16	π	C7–C8	π*	20.00	0.29	0.069
	C18–C19	π	C1–C2	π*	19.65	0.28	0.068
	N23	LP(1)	C1–C2	π*	28.3	0.31	0.089
	O24	LP(2)	C16–C17	σ*	21.11	0.7	0.11
	O24	LP(2)	C17–C18	σ*	21.99	0.68	0.11
2-Br-9-Fl	C4–C11	π	C5–C6	π*	21.03	0.29	0.07
	C4–C11	π	C8–C10	π*	19.72	0.27	0.065
	C5–C6	π	C8–C10	π*	23.28	0.27	0.071
	C8–C10	π	C4–C11	π*	20.08	0.31	0.07
	C13–C21	π	C14–C15	π*	21.59	0.29	0.071
	C14–C15	π	C17–C19	π*	21.12	0.28	0.07
	C17–C19	π	C13–C21	π*	20.73	0.29	0.07
	Br 1	LP (3)	C8–C10	π*	9.81	0.3	0.053
	O2	LP (2)	C3–C4	σ*	21.14	0.69	0.109
	O2	LP (2)	C3–C13	σ*	20.58	0.69	0.108
2-OH-9-Fl	C3–C4	π	C12–C13	π*	21.69	0.27	0.07
	C5–C7	π	C3–C4	π*	20.4	0.29	0.069
	C6–C8	π	C14–C15	π*	21.75	0.28	0.071
	C10–C11	π	C6–C8	π*	21.72	0.29	0.071
	C12–C13	π	C5–C7	π*	20.14	0.3	0.07
	C14–C15	π	C10–C11	π*	20.95	0.29	0.07
	O1	LP (2)	C3–C9	π*	20.92	0.69	0.108
	O1	LP (2)	C9–C10	σ*	20.47	0.7	0.108
	O2	LP (2)	C12–C13	π*	28.84	0.34	0.095

### 3.5 Surface Analysis

The fluorenone derivatives for their electrostatic potential (ESP) and Average Local Ionization Energy (ALIE) values were analyzed using Multiwfn software. The resulting surface maps were visualized with VMD software and are presented in Figure 6. In the ESP maps, blue corresponds to the lowest electrostatic potential values, and red represents the highest values. As observed in the ESP surfaces of these molecules, compatible with the literature, the most negative potential is concentrated on the carbonyl group of the cyclopentadienone ring.^50^ These regions are electron-rich and therefore attract electrophiles. In 2-nitro- and 2-hydroxy-9-fluorenone molecules, the negative electrostatic potential is also localized on the oxygen atoms of the respective substituents. The positive electrostatic potential regions (red areas) are concentrated on the hydrogen atoms in all four molecules, representing electron-poor regions that are favorable for nucleophilic attacks. Overall, it is evident that different substituents not only influence the ESP distribution in their immediate vicinity but also modulate the electrophilic and nucleophilic character of the entire fluorenone framework. ALIE calculations were performed to examine regions on the molecular surfaces where electrons are more loosely or tightly bound, and the results are also shown in Figure 6. ALIE surfaces provide an effective way to evaluate the susceptibility of molecules to electrophilic and nucleophilic attacks, and the blue regions in the maps correspond to areas where electrons are less tightly bound and thus more sensitive to electrophilic attack. Cyan-colored regions indicate the minimum average ionization energy (I̅). The color scale ranges from blue to white to red, with blue representing regions of weakest electron binding.^51^ As observed in Figure 6, the nitro, hydroxy, amino, and bromo substituent groups affect the electronic structure of the fluorenone ring and also cause changes in the local ionization energy along the ring.

**Figure 6:**
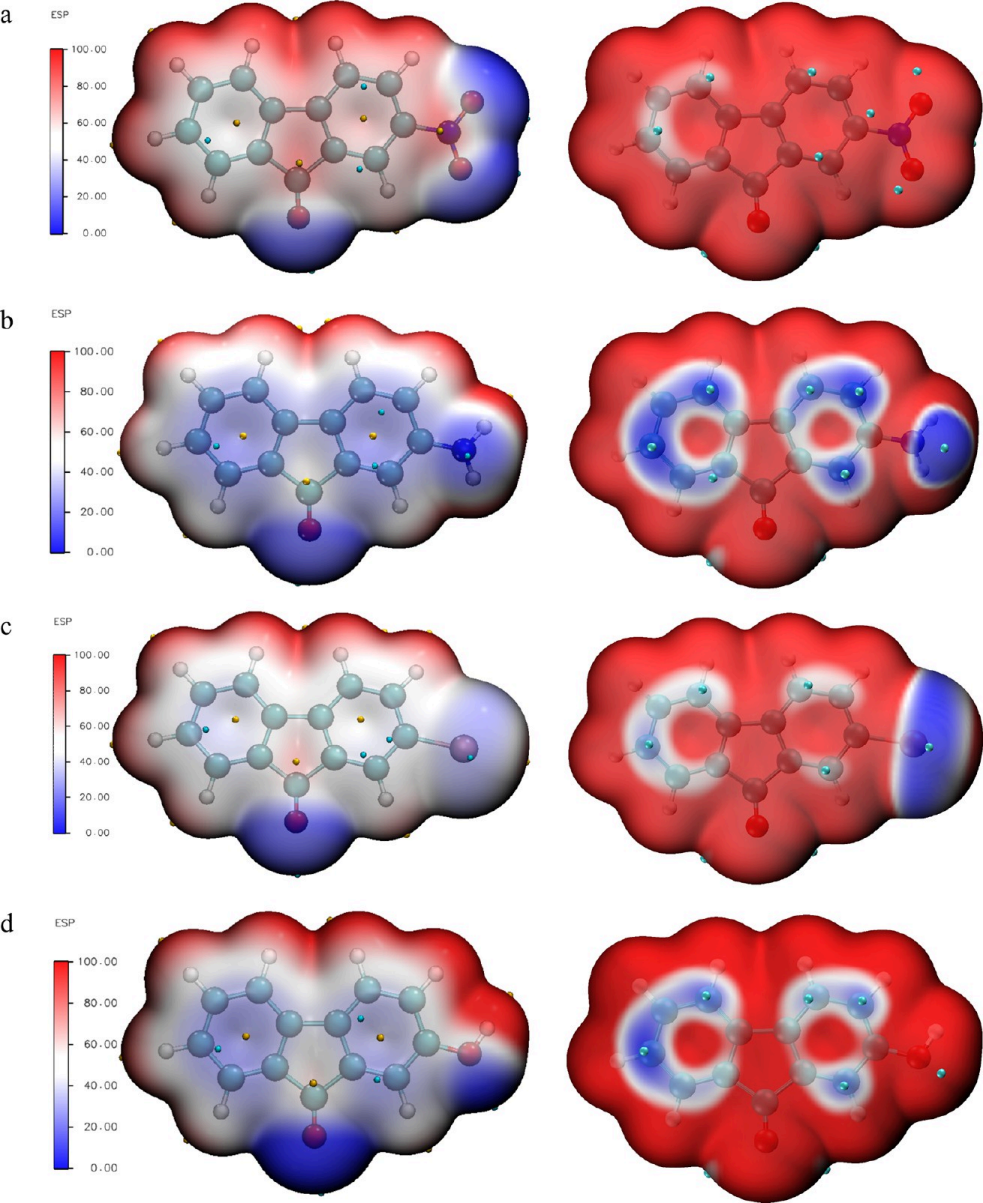
Surface maps of 2-NO_2_-9-Fl (a), 2-NH_2_-9-Fl (b), 2-Br-9-Fl (c), and 2-OH-9-Fl (d) determined using Mutiwfn.

### 3.6 Absorbance and Emission Study

The electronic transition properties of fluorenone derivatives were investigated through calculations performed using the TD-DFT method, the long-range corrected DFT functional, CAM-B3LYP^52^ and the 6–311++G­(d, p) basis set. In the excited state analysis, six excited states (*n*states = 6) were calculated, with the first excited state (root = 1) serving as the basis for the evaluations. The dispersion effects were defined using the GD3 empirical dispersion correction,^53^ and the solvent effect was defined using the CPCM model and dichloromethane (DCM) was selected as the solvent. The absorption and emission spectra were obtained at the same TD-DFT level and are shown in Figure 7. The absorbance and emission data are given in Tables 4 and 5, respectively. As illustrated in Table 4, the absorption energies, oscillator strengths, wavelengths, and main molecular orbital contributions corresponding to the first excitation and highest oscillator strength electronic transitions for each of the 2-NO_2_-9-Fl, 2-NH_2_-9-Fl, 2-Br-9-Fl and 2-OH-9-Fl in DCM are presented. It has been observed that the maximum absorption bands of 2-nitro-, 2-amino-, 2-bromo-, and 2-hydroxy-9-fluorenone appear at 278.16 nm (4.46 eV), corresponding to the HOMO–1 → LUMO (%46) transition, at 260.13 nm (4.77 eV), corresponding to the HOMO–1 → LUMO (%55) transition, at 248.35 nm (4.99 eV), corresponding to the HOMO → LUMO+1 (%59) transition, and at 249.77 nm (4.96 eV), corresponding to the HOMO → LUMO+1 (%53) transition, respectively. For each derivative (2-NO_2_-9-Fl, 2-NH_2_-9-Fl, 2-Br-9-Fl and 2-OH-9-Fl), the first singlet excitation (S0→S1) is primarily related to the HOMO → LUMO transition and can be considered as the calculated optical band gap (Eopt) of the relevant molecule. The first lowest-energy electronic excitations occur at 3.42 eV (362.11 nm), 2.87 eV (432.57 nm), 3.31 eV (374.96 nm) and 3.11 eV (398.73 nm) respectively, and primarily correspond to the HOMO → LUMO transitions (76, 96, 95 and 97%) of each molecule. According to the results, the absorption spectra show a slight red shift in the order: 2-bromo < 2-hydroxy < 2-amino < 2-nitro 9- fluorenone. These bands are typically indicate π–π* transitions or *n*-π* transitions within the studied molecules.^54^ The emission from the excited state to the ground state was calculated by using the same procedure as that used for absorption, corresponding to the fluorescence process (*E*_fluo_), but based on the optimized excited state geometry. The emission spectra were calculated at the CAM-B3LYP/6–311++G (d,p) level using TD-DFT with Grimme’s GD3 empirical dispersion and the CPCM solvent model (solvent = DCM) and shown in Figure 7. The data were listed in Table 5. According to Table 5, the calculated emission energies for 2-nitro-, 2-amino-, 2-bromo-, and 2-hydroxy-9-fluorenone correspond to S5 → S0 transitions. 2-nitro-9-fluorenone, exhibits an emission of 4.37 eV (283.79 nm), primarily associated with the H-1 → L transition (52%), while 2-amino-9-fluorenone, emits at 4.62 eV (268.26 nm) associated with H-2 → L (37%), 2- bromo-9-fluorenone is associated with 4.84 eV (256.08 nm) associated with H → L+1 (52%), and 2-hydroxy-9-fluorenone is associated with 4.81 eV (257.69 nm) associated with H → L+1 (44%). These results demonstrate that the UV–vis absorption properties of fluorenone derivatives can be accurately predicted using TD-DFT and that the obtained data are compatible with literature values for fluorenone and its derivatives. The literature indicates that the absorption bands of fluorenone (FL) are 265–310 nm (weak) and approximately 257 nm (strong) in nonpolar solvents, 285 nm in MCH and acetonitrile, and around 288 nm in benzene. Similarly, 1-hydroxyfluorenone (1HOF) shows absorption at 265–325 nm (weak) and approximately 260 nm (strong), and 3-dimethylaminofluorenone (3DMAF) is known to exhibit absorption in the 300–350 nm (weak) and approximately 280 nm (strong) regions. Furthermore, upon examination of the fluorescence spectra, bands at approximately 310 nm (monomer) and approximately 460 nm (excimer) for FL, approximately 322 nm (monomer) and approximately 480 nm (excimer) for 1HOF, and approximately 350 nm (monomer) and approximately 570 nm (excimer) for 3DMAF have been reported. Therefore, the consistency of the data obtained in our study with the absorption and fluorescence bands reported in the literature supports the reliability and predictive power of the calculation methods used.^14,55^

**Figure 7:**
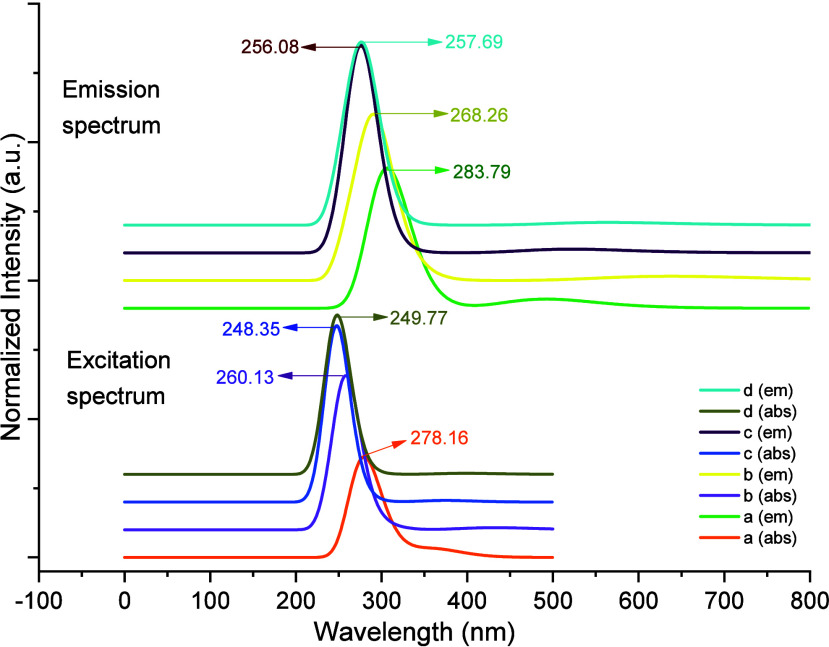
Emission and excitation spectrum of 2-NO_2_-9-Fl (a), 2-NH_2_-9-Fl (b), 2-Br-9-Fl (c) and 2-OH-9-Fl (d) in CPCM/DCM.

**Table 4:** Computed Absorbance Parameters of the Fluorenone Derivatives

molecules	electronic transition	*f*	*E* (eV)	λ (nm)	^a^major contribution (%)
2-NO_2_-9-Fl (a)	S_0_→S_1_	0.0740	3.4239	362.11	H→L (76%)
	S_0_→S_5_	0.7535	4.4573	278.16	H-1→L (46%)
2-NH_2_-9-Fl (b)	S_0_→S_1_	0.0187	2.8662	432.57	H→L (96%)
	S_0_→S_5_	1.2228	4.7663	260.13	H-1→L (55%)
2-Br-9-Fl (c)	S_0_→S_1_	0.0145	3.3066	374.96	H→L (95%)
	S_0_→S_5_	1.4046	4.9924	248.35	H→L+1 (59%)
2-OH-9-Fl (d)	S_0_→S_1_	0.0097	3.1095	398.73	H→L (97%)
	S_0_→S_5_	1.2263	4.9639	249.77	H→L+1 (53%)

a Only the major (highest) orbital contribution is shown; other transitions also contribute.

**Table 5:** Computed Emission Parameters of the Fluorenone Derivatives

molecules	electronic transition	*f*	*E* (eV)	λ (nm)	^a^major contribution (%)
2-NO_2_-9-Fl (a)	S_5_→S_0_	0.8046	4.3689	283.79	H-1→L (52%)
2-NH_2_-9-Fl (b)	S_5_→S_0_	0.7508	4.6219	268.26	H-2→L (37%)
2-Br-9-Fl (c)	S_5_→S_0_	1.4728	4.8417	256.08	H→L+1 (52%)
2-OH-9-Fl (d)	S_5_→S_0_	1.2355	4.8114	257.69	H→L+1 (44%)

a Only the major (highest) orbital contribution is shown; other transitions also contribute.

### 3.7 InterFragment Charge Transfer (IFCT) and Charge Transfer Matrix (CTM)

Charge transfer between fragments is a crucial event in the electron excitation processes. The IFCT method in Multiwfn software developed by Tian Lu^56^ provides numerical values of the charge transfer occurring between fragments used in this study. To characterize the charge transfer properties of fluorenone derivatives under excited state conditions, we performed IFCT and CTM analyses were performed. With IFCT, the amount of charge transfer (QCT) occurring between fragments in a molecule during electron excitation was calculated and is shown in Table 6. Determining the direction of charge transfer between molecular fragments is crucial for understanding the charge transfer (CT) transition properties of the material.^57,58^ Therefore, this direction, as seen in Table 6, for 2-NO_2_–9-Fl (S0→S5), the intrafragment electron redistribution of fragments 1 (fluorenone) and 2 (nitro group) was measured as a net electron transfer of 0.23935 electrons from fragment 1 to fragment 2.

**Table 6:**
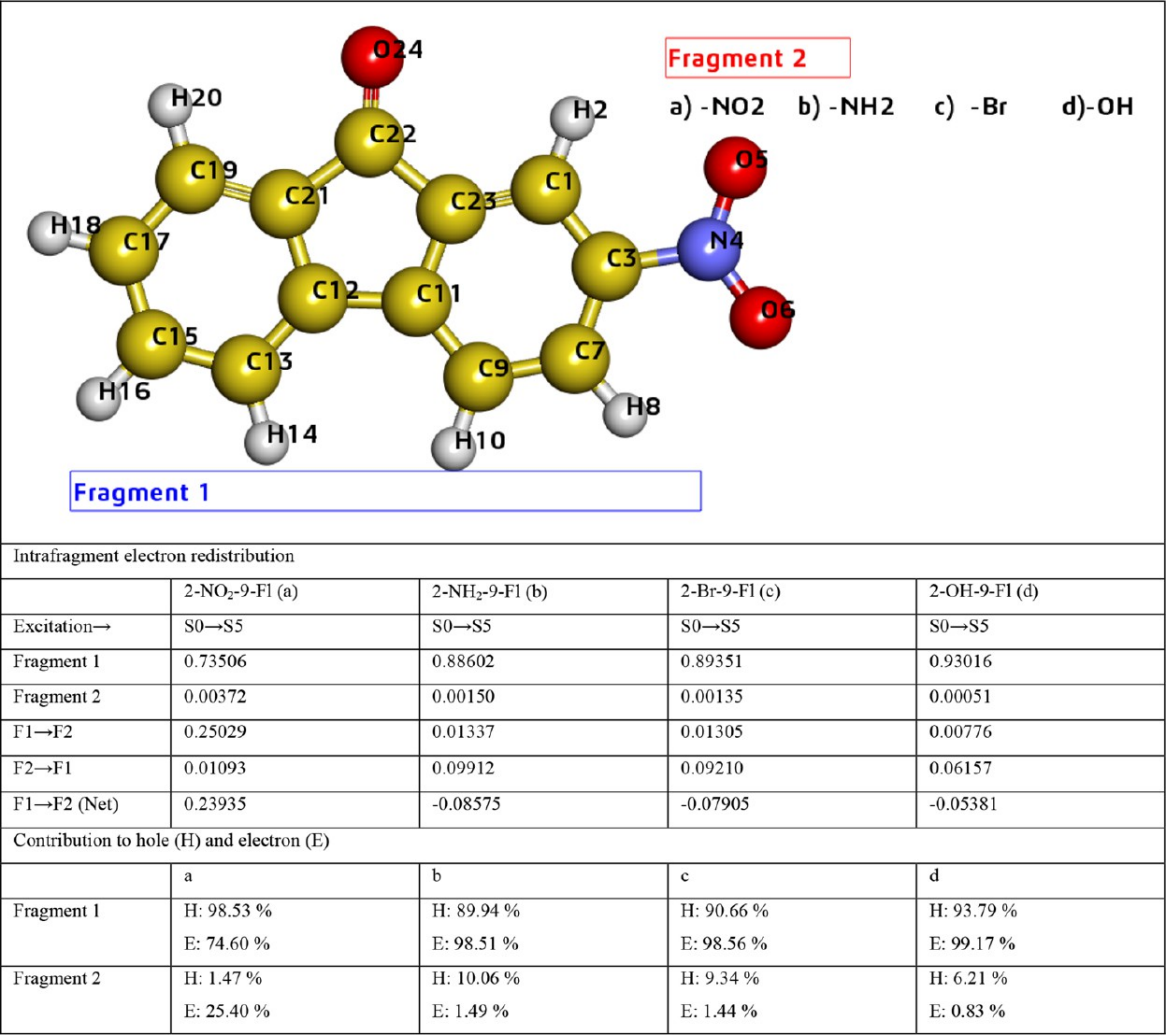
Interfragment Charge Transfer (IFCT) Analysis for the Fluorenone Derivatives

The atom–atom charge transfer matrix, known as CTM, is used to determine the direction of charge transfer during electron excitation. The charge transfer matrix (CTM), derived within the theoretical framework of hole–electron analysis, can better reveal the actual charge transfer character.^57,58^ In a CTM, each off-diagonal element represents the amount of electron transfer between atoms and each element (Ai, Aj) corresponds to charge transfer from atom Aj (hole) (*x*-axis) to atom Ai (electron) (*y*-axis).^57^ For instance, in Figure 8a, the matrix element (22,13) serves as an example of charge transfer from atom 13 to atom 22 and the related density value indicates the density of the transfer. Moreover, in Figure 8a, the value in the matrix element (22,13) is higher than the values in the matrix elements (3,13), (7,13), (11,13), indicating that electron transfer from C13 to C22 is greater than transfer to other atoms. The other transitions can be examined in Figure 8a. In Figure 8b, the matrix element (17,16) shows a distinctly higher value, indicating that electron transfer from C16 to C17 is dominant. In Figure 8c, electron transfers from C4 to C6, from C4 to C11, and from C11 to C6 are particularly prominent. Furthermore, this bromine-substituted fluorenone derivative exhibits brighter yellow highlights, indicating that electron transfer occurs between a greater number of atoms. Finally, in Figure 8d, the transfer from C4 to C7 is observed to be more pronounced.

**Figure 8:**
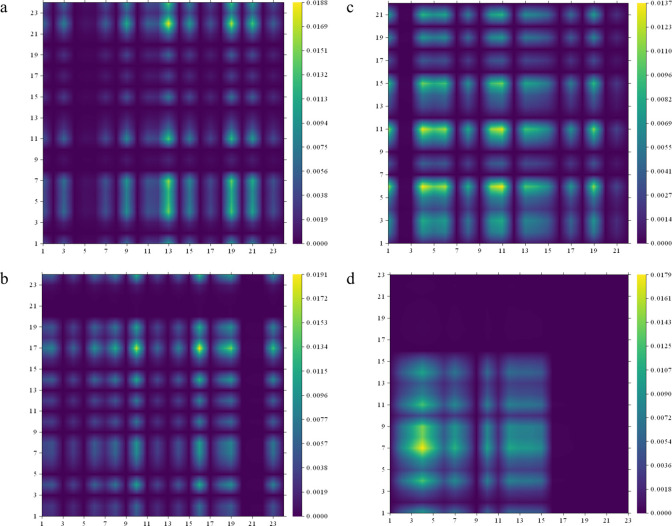
Charge transfer matrix (CTM) of 2-NO_2_-9-Fl (a), 2-NH_2_-9-Fl (b), 2-Br-9-Fl (c), and 2-OH-9-Fl (d).

### 3.8 LOLIPOP and HOMA Analysis

In this study, LOLIPOP calculations were performed based on the Localized Orbital Locator-π (LOL-π) approach.^59^ Using π-LMOs automatically determined by the Multiwfn program, LOL-π isosurface maps were created with a 0.5 isosurface value to visualize π-delocalization pathways. For each fluorenone derivative, the LOLIPOP values calculated for the aromatic ring to which the substituent is attached and for the other aromatic ring were determined and are presented in Figure 9. According to Figure 9, the LOLIPOP index indicates that the aromatic rings belonging to bromine and hydroxy substituted fluorenone rings have the lowest values, which are 2.399 and 3.071, and 4.307 and 3.195, respectively, and therefore have the strongest π–π stacking potential. In contrast, the nitro-substituted ring exhibits higher LOLIPOP values of 6.104 and 4.382, and the amino-substituted ring exhibits higher LOLIPOP values of 5.085 and 4.233, indicating a relatively weaker π–π interaction potential. That is, the low LOLIPOP values obtained here indicate that the molecule may exhibit relatively stronger π–π interactions and, consequently, may have better π-stacking ability,^60^ and it serves as a tool that can be used to screen these potential π-stacking candidates, particularly in the context of chemosensor design.^59^ Furthermore, the Harmonic Oscillator Model of Aromaticity (HOMA) was employed to assess aromaticity. HOMA is one of the most widely used indices for measuring aromaticity.^61^ The HOMA index for benzene rings (yellow and purple rings as highlighted in Figure 9) was found to be 0.9717 and 0.9693 for 2-NO_2_-9-Fl, 0.9483 and 0.9643 for 2-NH_2_-9-Fl, 0.9744 and 0.9683 for 2 -Br-9-Fl, and 0.9628 and 0.9668 for 2-OH-9-Fl, respectively. If the HOMA value is 1, the ring is completely aromatic; if the HOMA value is 0, the ring is not completely aromatic. If the HOMA value is significantly negative, the ring exhibits antiaromatic properties.^61^ Since the HOMA values are very close to 1, it can be said that the molecules examined possess aromaticity.

**Figure 9:**
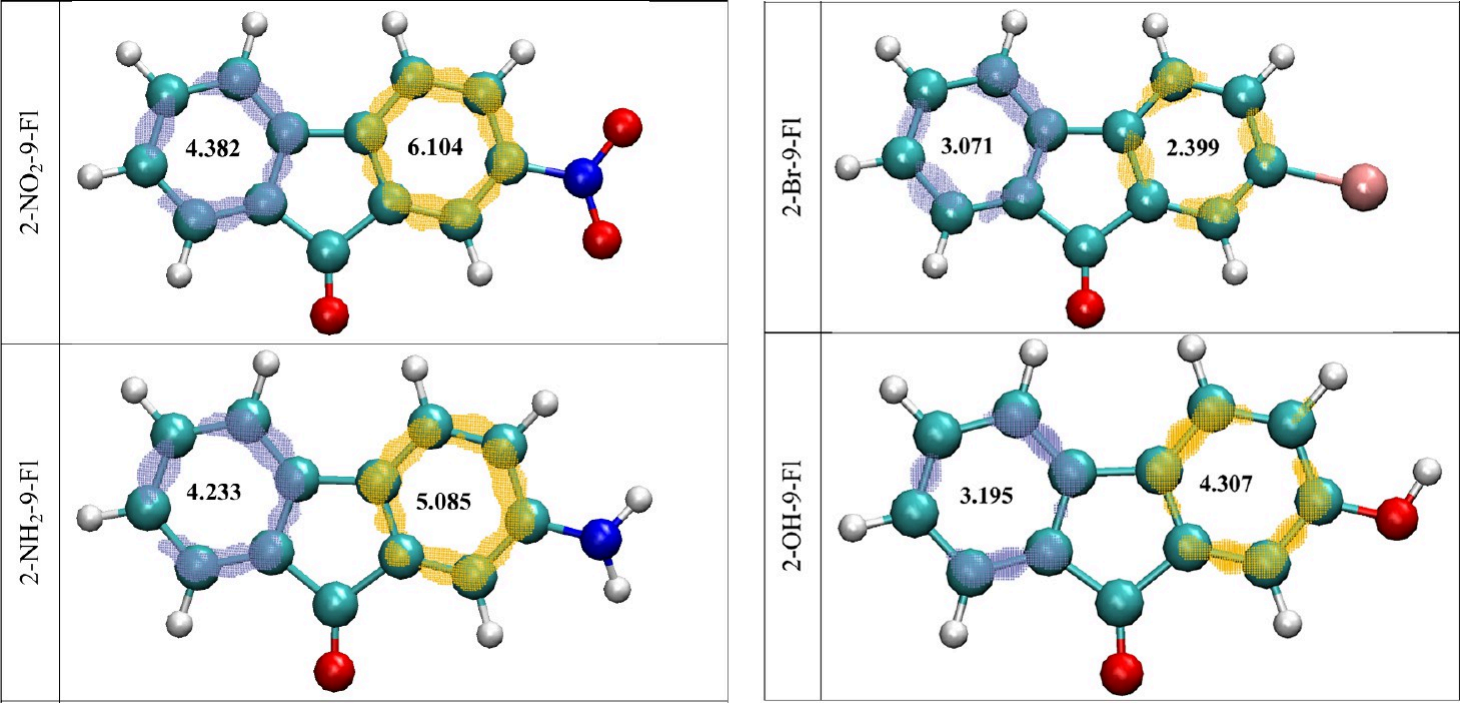
LOLIPOP index obtained from expected π-orbitals (isovalue of 0.5) of 2-NO_2_-9-Fl (a), 2-NH_2_-9-Fl (b), 2-Br-9-Fl (c), and 2-OH-9-Fl (d).

### 3.9 NLO Analysis

Research on the nonlinear optical properties (NLO) of organic substances have become popular in recent years due to their significance in photonic applications.^62^ To understand the optical properties, the (hyper) polarizability parameters of 2-nitro-, 2-amino-, 2-bromo-, and 2-hydroxy-9-fluorenone derivatives were calculated at different frequencies, and the obtained data are presented in Table 7. In this study, the average linear polarizability (α_0_), the total static dipole moment (μ), the polarizability anisotropy (Δα), and the first-order hyperpolarizability (β) were determined by Multiwfn and DFT (CAM-B3LYP-D3). This analysis provides important information about how substituents affect the electronic distribution of molecules and, consequently, their nonlinear optical responses. The static total dipole moment (μtot) values for 2-nitro-, 2-amino-, 2-bromo-, and 2-hydroxy-9-fluorenone were found to 7.25 × 10^–18^, 3.84 × 10^–18^, 4.37 × 10^–18^, and 4.93 × 10^–18^ D. The 2-nitro-9-fluorenone (a) has the highest μtot value. Table 7 shows that the average polarizability (α_0_) and polarizability anisotropy (Δα) values also exhibit differences depending on the effect of the substituent. Fluorenone’s 2-nitro- and 2-bromo- derivatives generally exhibit higher α_0_ and Δα values compared to 2-amino- and 2-hydroxy- derivatives. Under static conditions, α_0_ and Δα values for all derivatives increase with increasing frequency (0.05, 0.07, and 0.1), and both α_0_ and Δα values increase in under dynamic conditions. For the nonlinear optical parameter βtot values; in the static state: 1.31 × 10^–29^, 1.09 × 10^–29^, 2.92 × 10^–30^, 6.49 × 10^–30^ esu; when the frequency is 0.05 (911 nm); 1.67 × 10^–29^, 1.30 × 10^–29^, 3.50 × 10^–30^ and 7.44 × 10^–30^. The values for other frequencies are listed in Table 7. The 2-nitro- substituent of 9-fluorenone exhibits the highest first-order hyperpolarizability, while the 2-bromo- substituent of 9-fluorenone has the lowest value. As the frequency increases, βtot increases, and particularly at 0.10 au (455.63 nm), the βtot value in the nitro derivative is 4.24 × 10^–29^ esu. Urea, a well-known nonlinear optical (NLO) reference material, exhibits a first hyperpolarizability (β) values of 0.066 × 10^–30^ esu, calculated at the CAM-B3LYP/6–311++G­(d,p) level of theory in CH_2_Cl_2_.^63^ In comparison, 2-nitro-9-fluorenone shows a first hyperpolarizability approximately 198.5 times higher than that of urea calculated. From the above results, it is clear that the electronic distribution is affected by the type of substituent and has an effect on NLO response properties.

**Table 7:** Calculated (Hyper)­polarizability Values for the Fluorenone Derivatives[Table-fn t7fn1]

parameter	a	b	c	d
Static (0.00)				
μtot(D)	7.245693 × 10^–18^	3.844614 × 10^–18^	4.374320 × 10^–18^	4.926044 × 10^–18^
α_0_ (esu)	2.574631 × 10^–23^	2.476379 × 10^–23^	2.611050 × 10^–23^	2.346910 × 10^–23^
Δα (esu)	2.392973 × 10^–23^	2.216190 × 10^–23^	2.409619 × 10^–23^	2.038265 × 10^–23^
βtot (esu)	1.310073 × 10^–29^	1.085293 × 10^–29^	2.922693 × 10^–30^	6.485312 × 10^–30^
βprj	1.168149 × 10^–29^	4.701467 × 10^–30^	–7.415921 × 10^–31^	4.561376 × 10^–31^
β∥	7.008892 × 10^–30^	2.820880 × 10^–30^	–4.449553 × 10^–31^	2.736825 × 10^–31^
β∥<*z*>	4.425389 × 10^–35^	–1.640654 × 10^–31^	5.839036 × 10^–35^	1.231248 × 10^–35^
β⊥<*z*>	1.475130 × 10^–35^	–5.468846 × 10^–32^	1.946345 × 10^–35^	4.104160 × 10^–36^
Dynamic (0.05, 911.27 nm)				
α_0_ (esu)	2.654412 × 10^–23^	2.551423 × 10^–23^	2.682779 × 10^–23^	2.410727 × 10^–23^
Δα (esu)	2.537526 × 10^–23^	2.347286 × 10^–23^	2.534621 × 10^–23^	2.142142 × 10^–23^
βtot (esu)	1.667975 × 10^–29^	1.303680 × 10^–29^	3.498918 × 10^–30^	7.448321 × 10^–30^
βprj	1.482129 × 10^–29^	5.658666 × 10^–30^	–8.662105 × 10^–31^	6.347345 × 10^–31^
β∥	8.892773 × 10^–30^	3.395200 × 10^–30^	–5.197263 × 10^–31^	3.808407 × 10^–31^
β∥<*z*>	5.261244 × 10^–35^	–1.899409 × 10^–31^	6.299030 × 10^–35^	1.493955 × 10^–35^
β⊥<*z*>	1.775933 × 10^–35^	–6.519119 × 10^–32^	2.146444 × 10^–35^	5.091206 × 10^–36^
Dynamic (0.07, 650.91 nm)				
α_0_ (esu)	2.743041 × 10^–23^	2.634782 × 10^–23^	2.760185 × 10^–23^	2.479733 × 10^–23^
Δα (esu)	2.704139 × 10^–23^	2.498419 × 10^–23^	2.673583 × 10^–23^	2.257977 × 10^–23^
βtot (esu)	2.155491 × 10^–29^	1.613907 × 10^–29^	4.247753 × 10^–30^	8.693780 × 10^–30^
βprj	1.906681 × 10^–29^	6.961805 × 10^–30^	–1.058945 × 10^–30^	8.438878 × 10^–31^
β∥	1.144009 × 10^–29^	4.177083 × 10^–30^	–6.353669 × 10^–31^	5.063327 × 10^–31^
β∥<*z*>	6.323068 × 10^–35^	–2.250207 × 10^–31^	6.859240 × 10^–35^	1.830611 × 10^–35^
β⊥<*z*>	2.161949 × 10^–35^	–7.958462 × 10^–32^	2.369698 × 10^–35^	6.492116 × 10^–36^
Dynamic (0.1, 455.63 nm)				
α_0_ (esu)	2.995539 × 10^–23^	2.892991 × 10^–23^	2.966402 × 10^–23^	2.665961 × 10^–23^
Δα (esu)	3.213638 × 10^–23^	3.013072 × 10^–23^	3.065968 × 10^–23^	2.591095 × 10^–23^
βtot (esu)	4.244018 × 10^–29^	3.932578 × 10^–29^	7.371583 × 10^–30^	1.425628 × 10^–29^
βprj	3.696797 × 10^–29^	1.643828 × 10^–29^	–2.165990 × 10^–30^	1.536358 × 10^–30^
β∥	2.218078 × 10^–29^	9.862966 × 10^–30^	–1.299594 × 10^–30^	9.218147 × 10^–31^
β∥<*z*>	1.027664 × 10^–34^	–4.499858 × 10^–31^	8.860842 × 10^–35^	3.298713 × 10^–35^
β⊥<*z*>	3.870856 × 10^–35^	–1.806276 × 10^–31^	3.105523 × 10^–35^	1.633716 × 10^–35^

* β∥<*z*>:the parallel component of β with respect to *Z* axis, Βprj: the projection of β on dipole moment vector μ, β∥: is the β component in the direction of μ, β⊥<*z*>:the perpendicular component of β with respect to *z* axis.

### 3.10 Hirshfeld Surface Analysis and Crystal Packing Diagram

Hirshfeld surfaces are determined by a molecule’s proximity to its nearest neighbors and therefore provide crucial information for studying intermolecular interactions in crystals.^64^ To determine the HS interactions in fluorenone derivatives, the surfaces were analyzed using dnorm, di, fragment, and deformation density maps, and the results are presented in Figure 10.

**Figure 10:**
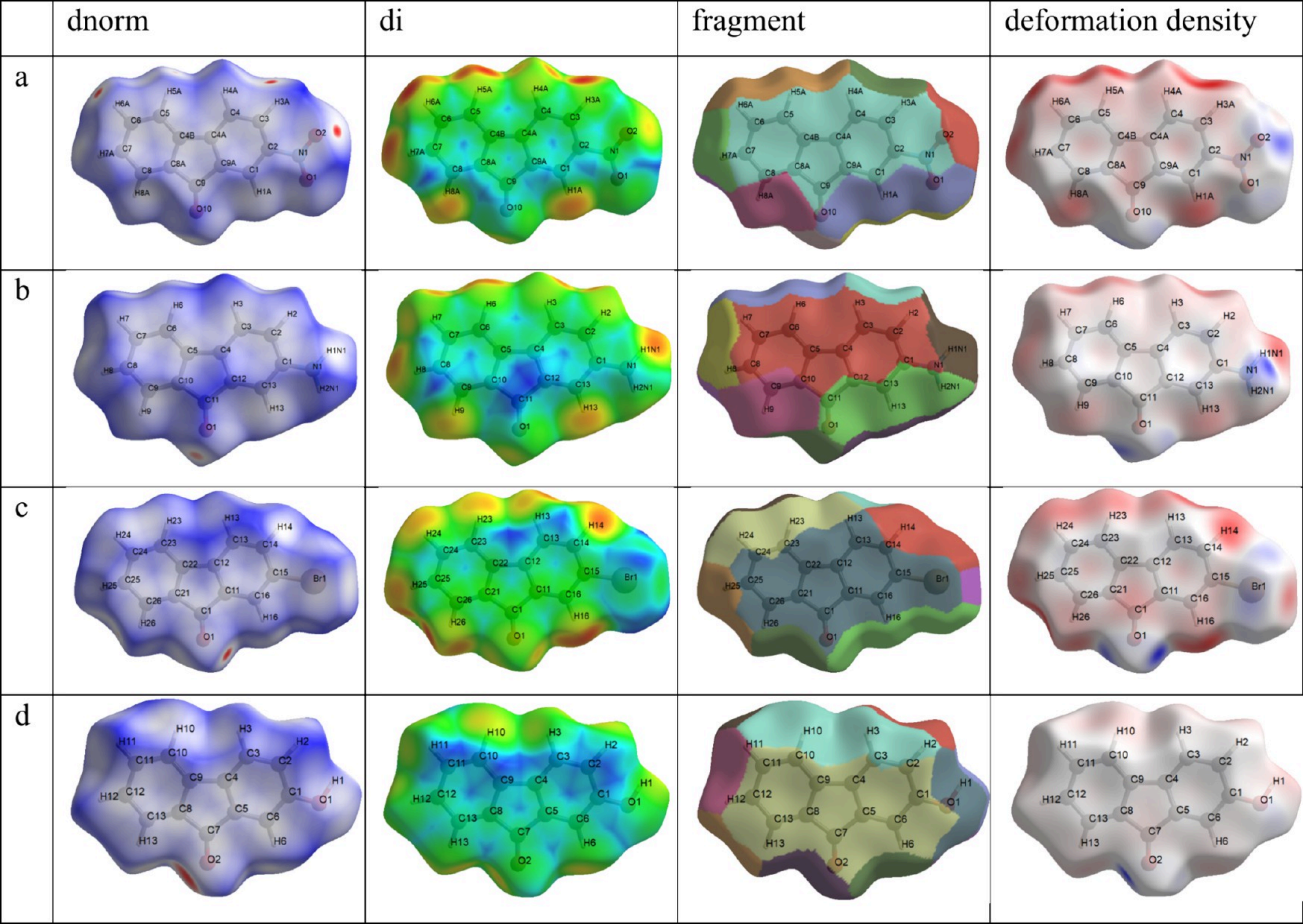
Hirshfeld surface for 2-NO_2_-9-Fl (a), 2-NH_2_-9-Fl (b), 2-Br-9-Fl (c) and 2-OH-9-Fl (d) mapped with *d*_norm_, *d*_*i*_, fragment, and deformation density.

To better visualize and interpret these interactions, a combination of d_i_ and d_e_ was employed in the form of a 2D fingerprint plot, as introduced by Spackman et al.,^64^ and the results are presented in Figure 11. As shown in Figure 11, changes in the substituent group affect the types of interactions and their percentages in the fingerplots. When the interactions that contribute to surface contacts in molecules are examined, it is seen that the O···H/H···O (internal and external) interactions, which constitute approximately 40% of the total contacts for 2-nitro-9-fluorenone (a), are predominant. These interactions are followed by H···H (21%), C···H/H···C (15.2%), and C···C (14.7%) contacts. In the 2-amino-9-fluorenone (b), 2-bromo-9-fluorenone (c), and 2-hydroxy-9-fluorenone (d) molecules, H···H contacts are predominant and contribute to surface interactions at rates of 45.4, 28.8, and 40.7%, respectively.

**Figure 11:**
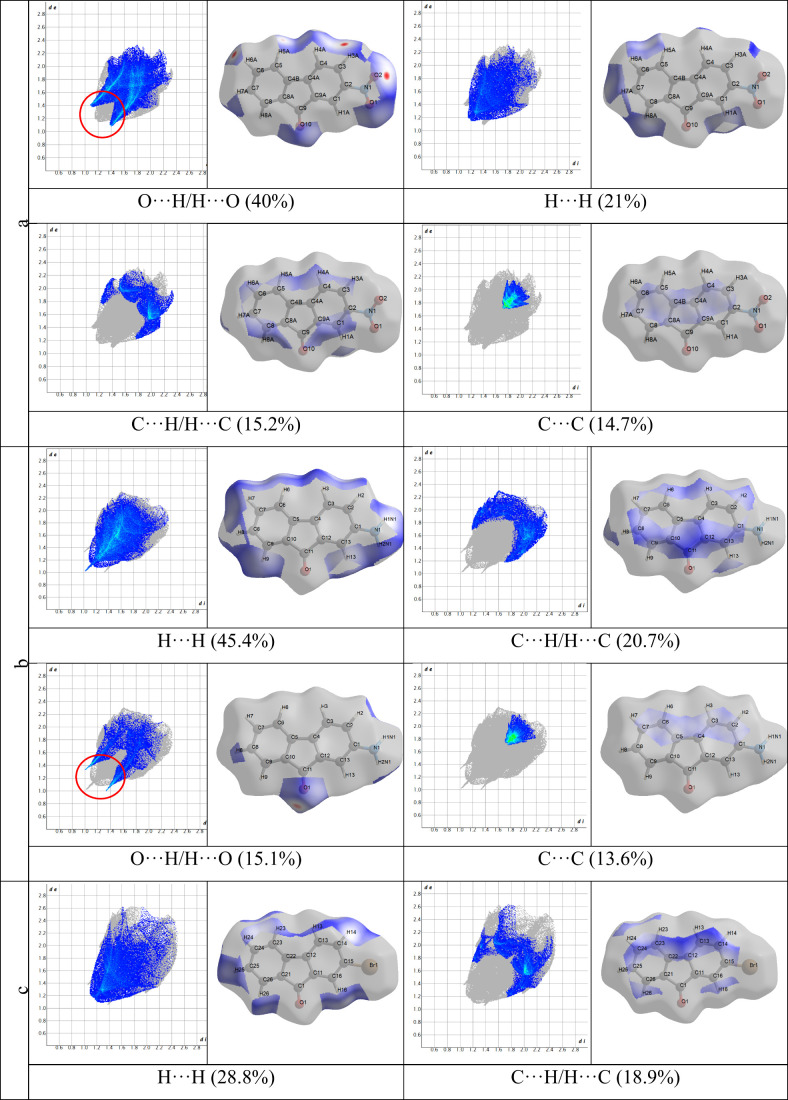

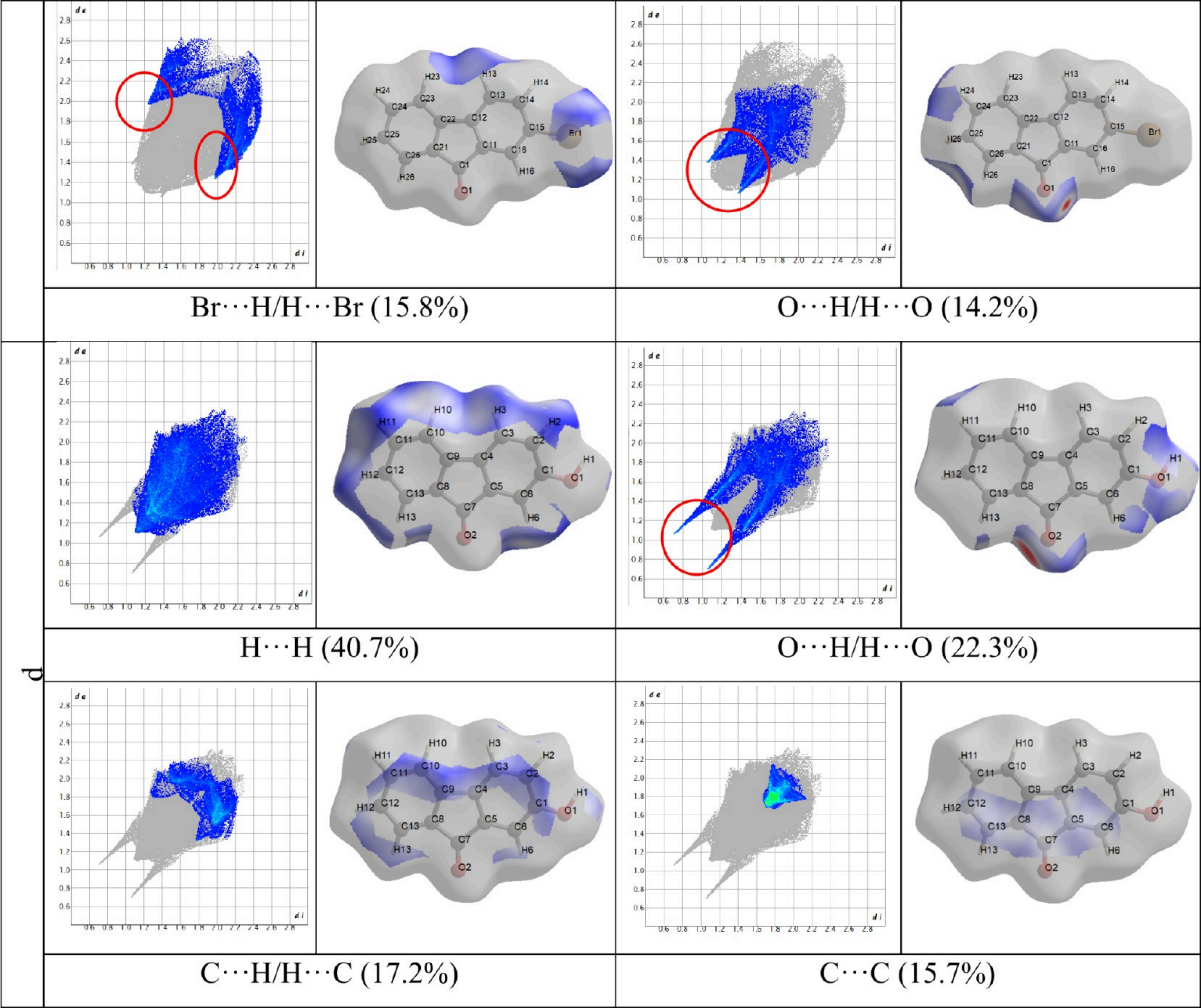
Fingerprint plots (the different types of interactions) for 2-NO_2_-9-Fl (a), 2-NH_2_-9-Fl (b), 2-Br-9-Fl (c), and 2-OH-9-Fl (d).

Additionally, to investigate both intramolecular and intermolecular interactions in fluorenone derivatives, crystal packing diagrams and bond lengths between atoms were analyzed by using the Mercury program, and the results are shown in Figure 12. It was observed that changes in the substituent groups attached to the fluorenone ring led to significant changes in these bond lengths. Among these, the interaction between the O1 and H12 atoms in 2-hydroxy-9-fluorenone stood out and showed a relatively stronger hydrogen bond (interaction highlighted with a blue box). This observation is further supported by a pair of sharp peaks in the H···O region of the fingerprint graph; these peaks are characteristic of stronger hydrogen bond interactions.^64^

**Figure 12:**
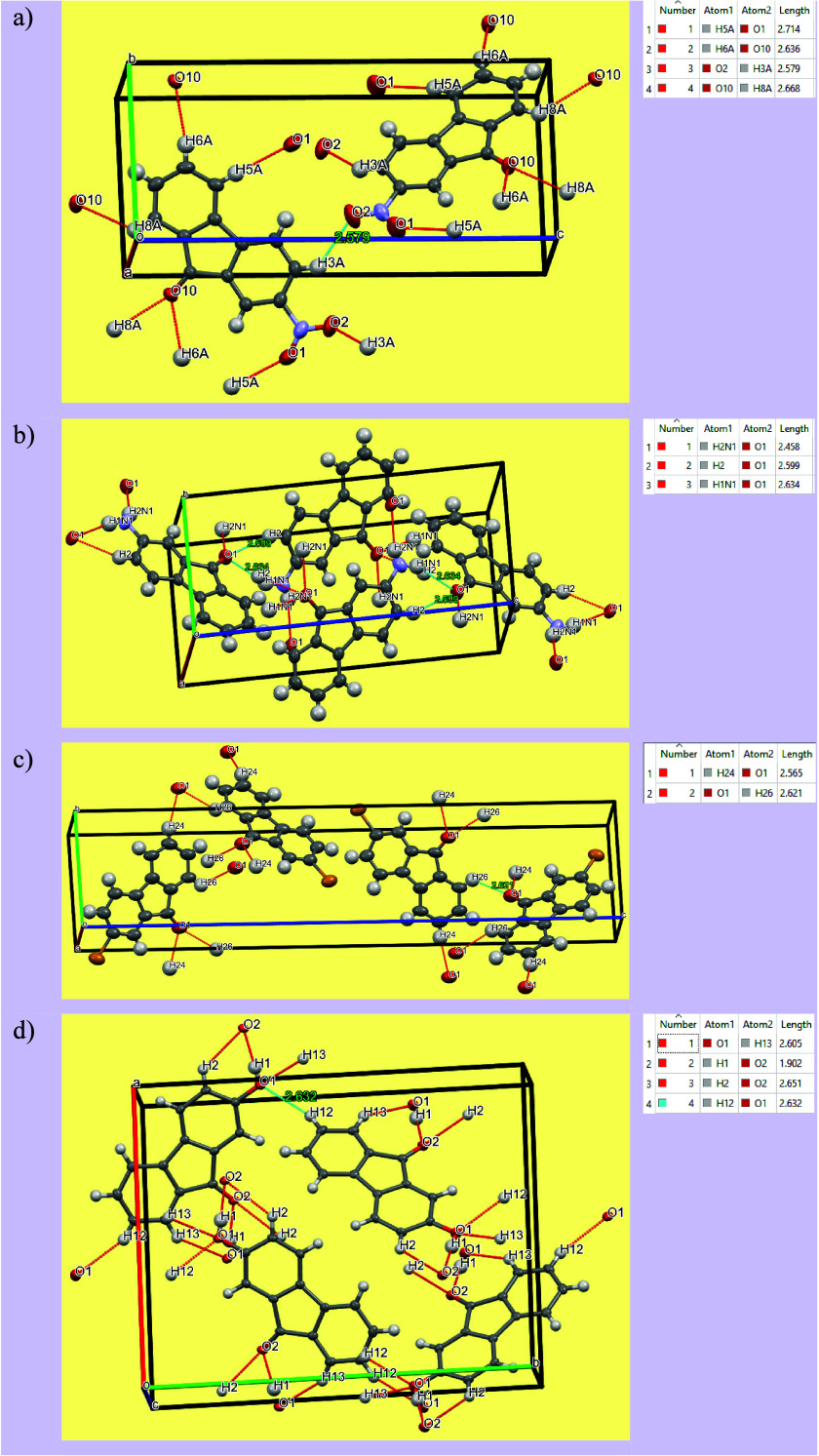
Crystal packing diagrams of 2-NO_2_-9-Fl (a), 2-NH_2_-9-Fl (b), 2-Br-9-Fl (c), and 2-OH-9-Fl (d).

### 3.11 ELF and LOL Analysis

The electron localization function (ELF), which describes the paired electron density, and the Localized orbital locator (LOL) analysis, which explains the maximum number of overlapping localized orbitals due to the orbital gradient, are two methods that have become widely used in recent years to characterize chemical bonds and identify electron-containing regions in atomic and molecular systems. LOL, like ELF, shows where electrons are concentrated, but it can provide clearer results, especially when dealing with complex bonds, transition metals, or multicenter structures. In this respect, it numerically supports intuitive models such as VSEPR, facilitating the interpretation of the structure and reactivity. Moreover, LOL stands out as a flexible and complementary bond analysis method, whether used in conjunction with ELF or on its own.^65^ The ELF and LOL maps of the fluorenone derivatives were drawn by using the Multiwfn program and are shown in Figure 13. In Figure 13, the red and orange colors on the maps indicate high electron localization, while the blue circle represents the region of low localization between the inner and valence shells. The red areas indicate highly localized electrons such as lone pairs, core electrons, or covalent bond electrons. The red regions around the hydrogen atoms indicate high ELF values and strong electron density in the bond regions. Blue areas around bromine, nitrogen, carbon and oxygen, on the other hand, indicate partially delocalized regions where electrons are less dense. The red regions between atoms reveal the presence of covalent bonds.^65,66^ In other words, the ELF primarily focuses on highlighting the positions of electron pairs including lone and bonding pairs. By contrast, the Localized orbital locator focuses on evaluating electron localization from an orbital perspective. This approach provides more direct insight into the character and structural details of the bond.

**Figure 13:**
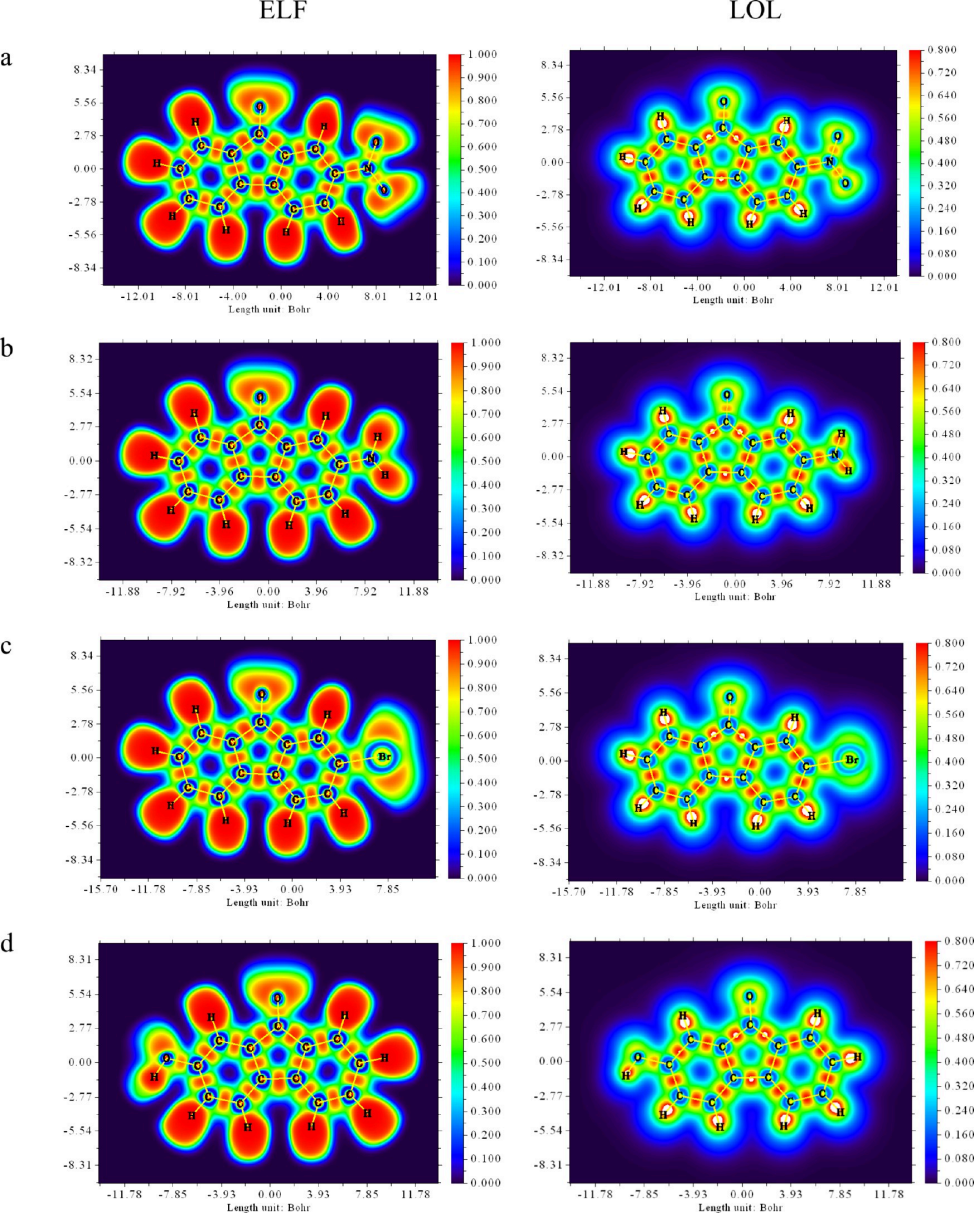
ELF and LOL analysis for 2-NO_2_-9-Fl (a), 2-NH_2_-9-Fl (b), 2-Br-9-Fl (c), and 2-OH-9-Fl (d).

## 4 Conclusion

In summary, four fluorenone derivatives were investigated to examine the effect of the substituents on the photophysical and electronic properties. The spectroscopic analyses revealed that electron-donating and -withdrawing substituents can be altered the results. Considering the main absorptions with the highest oscillator powers, the substituted fluorenone derivatives exhibit a gradual red shift in the S_0_→S_5_ transition in the order 2-bromo < 2-hydroxy < 2-amino < 2-nitro and this state is consistent with increasing λ values from 248 to 278 nm. NBO and IFCT analyses have shown that substituents affect the charge distribution of the molecule and charge transfer between fragments. It was found that the type of substituent affects the electronic distribution and influences the NLO response properties, with the greatest effect observed in the 2-nitro-9-florenone compound due to the electron-withdrawing effect. When examining the interactions within florenone derivatives, it was observed that 2-hydroxy-9-florenone molecules possess stronger hydrogen bonds than the others. The LOLIPOP index was determined, and it was found that the aromatic rings belonging to bromo- and hydroxy-substituted florenone rings had the lowest values. Therefore, they possessed the strongest π–π stacking potential. The HOMA index being close to 1 indicated that the molecules studied possessed aromatic properties. Crystal packing diagrams revealed that substituents affect bond lengths and intermolecular interactions, with 2-hydroxy-9-fluorenone exhibiting a notably strong O···H hydrogen bond, supported by fingerprint analysis.

## Data Availability

The data is available throughout the manuscript.
